# Supporting human-agent communication for explainable planning in spatial-temporal planning problems

**DOI:** 10.1007/s00521-025-11711-7

**Published:** 2026-05-06

**Authors:** Alan Lindsay, Andrés A. Ramírez-Duque, Bart Craenen, David A. Robb, Emanuele De Pellegrin, Laurence Boé, Andrea Munafò, Ronald P. A. Petrick

**Affiliations:** 1 Heriot-Watt University, Edinburgh, EH14 4AS UK; 2 SeeByte, Orchard Brae House, Edinburgh, EH4 2HS UK

**Keywords:** Explainable planning, Human-agent communication, Automatic model extensions, Transferable knowledge, Autonomous vehicles

## Abstract

The task of supporting a human operator to understand generated plans, and to explore the plan space, are important problems in automated planning. In this work, we consider the problem of plan explainability and plan space exploration in underwater autonomous vehicle missions. In this context, concepts that are useful for querying the system, such as distance and duration, will not necessarily map directly onto components of the planning model, such as actions. To overcome this difficulty, we focus on an important substructure of these problems: the multi-agent spatial-temporal (MAST) structure. Using this structure, we define a collection of model extensions, which include additional concepts relevant to the MAST structure. We then consider the problem of user-guided plan space exploration, and identify useful query types in this domain, including user queries based on numeric functions. These queries can make use of the extended model, allowing the user to directly reference the new concepts. In an empirical study, we demonstrate the use of the new structure within queries, and compare the new query types in our target domain, and in benchmark domains with the MAST structure. Finally, we report on a qualitative user study, where we investigate the use of these new structural concepts in underwater autonomous vehicle scenarios. Our study indicates that the extended concepts can be used in user queries and agent responses, enabling the user to better communicate their intent in shaping mission objectives, and supporting explanations with more relevant information.

## Introduction

The task of supporting human operators during planning of surveillance missions for autonomous underwater and surface robots is a challenging problem for human-agent interaction. In such missions, an operator must take into account various types of information, including topology, weather, robot capabilities, mission objectives and parameters, and safety requirements. This makes specifying the plan time-consuming and potentially error-prone. Furthermore, various competing factors must be considered when planning the mission, making it a process of trial and error.

This motivates the use of technologies such as Explainable AI Planning (XAIP) [1], which attempts to establish trust, interaction and transparency between users and AI controlled agents [2–5]. Contrastive queries and interaction have been identified as key components in supporting user understanding of AI based solutions [6], and there is already support to suggest that users find contrastive queries useful in plan-based approaches [7, 8]. For instance, the user might ask: *“Why does BlueROV1 transit from the launch to survey area 1?”* A contrastive explanation answers such a query by comparing the original plan with an alternative in which BlueROV1 does not make this transit, thereby highlighting the reason for its inclusion.

A common approach in XAIP, has been to consider that communication between the user and the system is based on structures in the planning model. In [7], actions are the building blocks, and the user can query the system’s choices about actions in the plan; in model reconciliation [9], the system considers a user model represented as a PDDL model; and, in [10], plan space explanation uses planning problem goals. In the example query above, such a query would be permitted in [7] only if a corresponding (transit BlueROV1 launch survey1) action were present in the planning model. Domain modellers must balance several factors when designing a domain model, including conciseness, appropriate plan language, solvability, and readability. When the user interrogates the system, their descriptions of the problem and solution will likely include references to structures that are not typically included in the planning model and include problem domain terminology [11]. As an illustration, consider the user query *“What is the total distance travelled by the vehicles in this plan?”* Here the query involves both collections of objects (the vehicles) and aggregate functions (the total distance travelled), elements that are generally not captured directly within the planning model.

In this work, we aim to address this gap between the planning model and the user’s conceptual model of the problem. Our approach relies on establishing a middle abstraction layer [4], which allows concepts to be shared between different problems, while also providing a suitable level for supporting knowledge to be expressed concisely and effectively. For instance, by specifying distance and its connections to planning actions in the middle layer, it becomes possible to represent derived concepts, including proximity and total distance travelled. Moreover, these concepts can be applied in other domains that involve spatial reasoning. This approach can be particularly suitable for automated planning, as many planning domains share concepts, such as spatial reasoning, navigation, and resource management [4]. This reduces the amount of work in creating domain-tailored content, while supporting new ways for the user to query the system, that is not supported by general approaches based on planning concepts.

We motivate this work with a scenario based on underwater autonomous vehicle (UAV) missions, and focus on the problem of effectively supporting human operators as they plan UAV missions. To motivate this problem, we focus on four example objectives that an operator might be considering when observing a plan for these tasks (see Subsect. 3.3), such as minimising the distance travelled by a vehicle to preserve an older battery, or avoiding high-risk areas. We identify an important structure within these problems: the multi-agent spatial-temporal (MAST) structure. Motivated by our UAV scenario, we identify MAST concepts that are important for reasoning in these scenarios, and notice that these are often missing from planning models (e.g., total distance travelled). For each of these concepts, we define a wrapper, which automatically extends the planning model with an additional model structure. We consider these extensions within the context of a plan explanation and exploration approach and define appropriate queries for user interaction in these problems. We present an empirical evaluation examining the use of the concepts in both UAV scenarios, and benchmark domains with MAST structures. Finally, we present a qualitative user study, which examines the use of the new structural concepts in UAV scenarios. The study indicates that the additional concepts allow the user to better shape mission objectives, better communicate their intent to the system, and extract more useful information from the explanations.

The contributions of this paper are as follows:

- We have conducted a user study investigating the question: ‘Does extending the concepts available for user queries allow for clearer communication with the system, and enable more useful explanations?’
- In Sect. 9 we present results indicating that the extended concepts can be used in user queries and agent responses, enabling the user to better communicate their intent in shaping mission objectives, and supporting explanations with more relevant information.
- In Sect. 4 we present a framework that establishes an abstraction layer, allowing concepts to be built from the planning model, interpreting aspects of the planning model structure, and supporting additional concepts that can be used in communication between the user and the system.
- In Sects. 5 and 6 we present an implementation of this abstraction layer through defining and exploiting the multi-agent spatial-temporal (MAST) structure. A key to our approach is that the concepts defined in Sect. 6 are available to be used with any planning domain with the MAST structure.
- In Sect. 7 we extend [7], and demonstrate how our approach can be used in the XAIP-as-a-service framework. We demonstrate how they can be used together in our UAV scenario.
- In Sect. 9 we present empirical results, demonstrating the applicability of the concepts defined in Sect. 6 in several MAST domains, while also demonstrating that the system generates alternative plans that are appropriate to input queries in those domains.
- In Sect. 8 we present a fully functioning XAIP toolkit that has been tested in planning an underwater robot mission in a real quarry.

Parts of this work appeared in 12 as a reduced presentation of the user study. That paper introduced a subset of the model extensions but did not include the supporting framework for applying them across domains, nor did it report any empirical results. The present article substantially extends that work by providing the complete framework, a broader set of extensions, empirical evaluation, and a deeper analysis of the study.

## Related work

### Human-aware and explainable planning

Human-aware planning is a growing field in automated planning, and examines the interaction between the user and the planning system, considering how to inform and support the user throughout the planning life-cycle, including modelling, planning, and executing. There are approaches that combine elicitation and planning/execution within a single framework, such as the factory setting, user tailoring, and execution tuning (FUTE) framework [13]. These frameworks are typically iterative, specialising plan generation through either interleaving elicitation and planning episodes [14], or learning from observations over time [13]. There are also more focused approaches, which examine generating explanations during specific stages, including the modelling process [15], planning [16], plans [7], and more general frameworks, e.g., [17], which provides a panel of alternative views on the developing information, including the plan.

Our work builds on research in the area of Explainable Planning (XAIP) [1]. This area has considered both the form and structure that the explanation should take [6, 18]. In [18] they investigated typical explanation structures, whereas in  [6] they provide insights into explanations from social sciences. They identified contrastive queries and interaction as key components in supporting user understanding of AI based solutions [6]. In the context of plan-based approaches, user studies have confirmed that users find contrastive queries for explanation and plan exploration useful [7, 8]. In [8] they present the results of a user study, where participants were tasked with optimising a plan while interacting with an iterative planning system. They demonstrate that a version supporting contrastive queries leads to improved performance. The approaches in [7, 10] each generate contrastive explanations, while supporting interactions. In these approaches the content of the explanations is generated using modifications to the planning model (see Sect. 3).

A growing number of approaches to XAIP have used visualisations [19–22] and these have proven successful in creating explanations [22]. There are now several alternative methods of plan visualising, e.g.,  [21] that operate from the planning model, plan and some form of metadata, which are used to parameterise the visualisation system. Using a similar approach, [22] generates explanations for model reconciliation and uses characterising traits of planning problems in order to simplify the specification of metadata. Existing work has also considered abstraction in XAIP, including verbalisation [23], visualisation [24], for knowledge representation [4]; and explanations for deployed robot systems, e.g., [25].

The problem of explanation has been set as a model reconciliation problem [9]. The problem from this viewpoint is that explanation is required because there is a difference between the human and agent models. There have been various approaches within this framework e.g., [9, 26], including generating explanations that suggest fixes to parts of the user’s model (e.g., include a missing precondition of an action) [9]. It is typical in these works to make assumptions about the user’s model. We accept there is a difference in models, but following [7], we do not make assumptions about what the human or agent knows about the other’s model. Instead, we follow the approach in [7], where any changes of the user’s model will arise from interacting with the agent, through a sequence of plan generation and user query steps. In contrast to the approach in [7], in which the user’s queries are tied to the actions in the planning model, we investigate bridging the human-agent representation gap, by introducing new concepts in an attempt to establish communication between the human and system at an appropriate level.

In [11] they consider existing work in terms of their desired criteria for bridging the representation gap between humans and robots. They focus mostly on learning approaches for individual problems. We instead consider developing a middle layer where structure can be exploited across several domains. We follow a similar framework to XAIP-as-a-service [7], which explains plans using contrastive explanations and allows users to explore plan space using queries. We have extended this approach in the context of UAV missions.

Much of the research on explanation in planning has focused on classical models. However, the demands of real-world domains have driven the development of explanation techniques for more expressive models [27], including temporal models [7], hybrid discrete–continuous models [27, 28], and models that capture uncertainty [20, 27]. In this work, we focus on temporal domains, allowing us to examine important causal and temporal concepts, which have been identified as central to how users query and interpret plans [7].

### Common model structures

It was observed in the early days of the international planning competition (ICP)^1^ that the majority of planning benchmarks could be grouped into common structures and themes [29], e.g., transportation (Driverlog etc.) and manipulation problems (Blocksworld etc.). The benchmark domains have continued to develop in complexity and diversity; however, key concepts such as traversal and resource management are still prevalent in planning domains, including real world applications, e.g, urban traffic control [30], underwater autonomous vehicle missions [31] and mining operations [32].

This commonality of structure in the benchmark problems has been exploited in planning in the planner, STAN [33], which decomposes planning problems around common subproblems, such as transportation. They demonstrate that common aspects of planning models such as transportation and resource management can be identified within the planning problem model’s structure. This has supported the effective description of specialised solutions to various problems including improving weak heuristics [34], inducing specialised heuristics [33], model extensions for control knowledge [35], selecting appropriate control knowledge [36], and generating plan explanations [4]. As in this work, a key benefit of the approach is that the solution can be specified at the level of the generic type or generic subproblem, but can be instantiated and exploited at the specific instance level.

### NLP, LLMs and planning

Our system includes a task-bot that maps user queries onto XAIP queries. Previous approaches disambiguating the mapping from natural language to planning models have relied on: a small set of training examples expressed in the target formalism [37], the use of commonsense knowledge [38], or learning the mapping for a specific domain, e.g., [39].

In the other direction, research has focused on generating natural language descriptions that convey model-relevant information to the user [23, 40]. In [23], the planning model is annotated with linguistic content, which is then used to construct suitable plan verbalisations. In [40], important concepts are abstracted from underlying feature vectors, with an attempt to fit meaningful lables to those abstractions. The intention in both cases is to assist the user in understanding the solution that the system has generated. By contrast, in our approach we first identify concepts that are useful to human operators and then support them explicitly through well-defined model extensions.

More recently LLMs have also become popular. However, due to sensitivity of the user data (e.g., the customers of the industry partner on our project include military and operators/technicians in the energy sector), it was not appropriate to consider their use in this work. Many applications in this field require rigorous adherence to repeatability standards, strict access controls, and robust security mechanisms. Unfortunately, relying exclusively on LLMs may not provide the necessary tools to meet these critical requirements, which are vital to maintaining the integrity and safety of command and control operations.

In recent years, LLMs have also become a popular approach for generating plans for robotics applications [41]. However, it is still not clear whether LLMs can be relied on to create executable plans, and how these approaches perform in domains that are outside the scope of their training set [42]. We adopt a plan-based approach, providing a predictable, robust and transparent process for creating plans, which supports our aims of assisting user understanding.

### Planning modelling support

In automated planning, modelling has been identified as a bottleneck, due to the skills required to develop these models. There are various approaches to support authoring PDDL models, including frameworks similar to IDEs for use by software engineers [43–45]. Alternatively, approaches have been developed that aim to reduce the burden of producing a complete domain model, including learning models from observations, e.g., [46–49], or queries [50], and providing assistance in refining [51] existing planning models. The main aim in these cases is to learn a planning model that captures the structure needed to effectively characterise valid trajectories in the problem domain. In contrast, we consider a complementary process: extending an existing representation with additional concepts, as needed, to support richer user queries and enable users to more precisely express their preferences or requirements for the trajectories. In  [52], they consider extending existing planning models. In that work, linguistic resources are combined with operations on the model structure to generate proposed model extensions. In our approach, we use structures within the planning model as the basis for extending the model.

## Background

In this section we define the planning formalism, we present the particular XAIP framework that we adopt in this work, and finally we introduce the autonomous vehicle scenario that motivate this work.

### Temporal planning problems

Following [53], a temporal planning problem can be defined as follows:

#### Definition 1

A Temporal Planning Problem is a planning problem, $P=\langle F,A,I,G,\mathcal {O},T\rangle$, with propositional and numeric fluents, *F*, actions, *A*, initial state, *I*, goals, *G*, $\mathcal {O}$ is the numeric objective function to be minimised, and a set of timed initial literals, *T*. An action, *a*, is defined by a duration (*dur*(*a*)), start ($cond_\vdash (a)$), invariant ($cond_\leftrightarrow (a)$) and end ($cond_\dashv (a)$) conditions, and start (*eff*$_{\vdash }(a)$) and end (*eff*$_{\dashv }(a)$) effects, which each describe the add and delete propositions, and numeric effects. Each $\langle p, t \rangle \in T$ specifies that literal *p* becomes true at absolute time $t \in \mathbb {R}_{0}^{+}$ during plan execution. A solution (a plan) is a schedule $\sigma$, which is a sequence of pairs $\langle a,t\rangle$, where $a\in A$ is an action, $t\in \mathbb {R}^{0+}$ is the action’s start time, *G* holds in the state after all actions have completed. For each pair in the schedule, we have action *a*, starting at time *t* (provided its start conditions are satisfied), and ending at time $t + dur(a)$ (provided its end conditions are satisfied), with any invariant conditions holding from *t* to $t + dur(a)$. The start effects of *a* are applied at *t*, and the end effects are applied at time $t + dur(a)$. Fluents and actions relate to a set of objects, *O* (e.g., locations, objectives, and vehicles).

The aim of temporal planning is to seek a plan that minimises the cost function $\mathcal {O}$, which unless stated we assume is to find a plan of short duration. A common language for specifying temporal planning models is PDDL2.2 [53, 54].

### XAIP-as-a-service

XAIP-as-a-service [7] is a framework that provides interactive access to planning systems, enabling users to pose queries and explore alternative solutions. This interactive model naturally supports explanations that are contrastive and selective, aligning with existing observations on what makes explanations effective [6]. In particular, [6] highlights that explanations are most useful when they address contrastive queries, focus on the most relevant causes, and occur within an interactive context. Building on these principles, contrastive explanation methods in planning, including [7], provide a concrete mechanism for generating explanations that are both targeted and interpretable. Our approach builds on the XAIP-as-a-service framework, and aims to support users to communicate their intent more clearly when shaping mission objectives and to receive explanations that provide relevant, actionable information. In this subsection we provide an overview of XAIP-as-a-service.

XAIP-as-a-service [7] is an approach within XAIP that operates from user queries about generated plans. The approach is based on taking the user’s query about a feature of a plan and generating a plan that exhibits the opposite feature. E.g., taking the query, *“Why is action A in the plan?”*, the approach generates a plan without action *A* and compares the plans empirically and structurally, demonstrating how the plan changes when action *A* is not used. In  [7] they consider queries including:

- Q1: *‘Why is A in the plan?’*
- Q2: *‘Why is A not in the plan?’*
- Q3: *‘Why is A used before B?’*

The approach relies on mapping user queries into constraints that can be added to the planning model, forcing any plans to exhibit the required property. For example in the case of Q1, a proposition *p* can be added to the initial state and removed when action *A* is used. In the goal we can add that *p* is required, forcing that *A* is never applied. The resulting constrained model is referred to as the hypothetical model (HModel), and any plan generated under these constraints as the hypothetical plan (HPlan). These terms reflect the idea that the user’s query is used to hypothesise an appropriate modification to the model in order to answer the query.

**Fig. 1:**
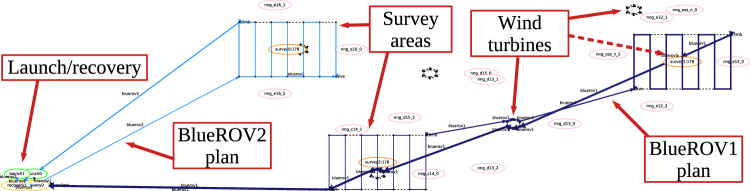
Illustration of a UAV survey mission with two UAV plans (light and dark blue), the launch/recovery, three survey areas, and six wind turbines.

### The underwater autonomous vehicle mission scenario

We adopt a case study based on underwater autonomous vehicle (UAV) missions [55, 56] to motivate and demonstrate our work. Existing research has considered the planning problem in the context of UAVs, including problem formulation [57], search strategies [58], and monitoring information opportunities during execution [59]. In our scenario, UAVs transit between waypoints, avoid areas that are dangerous to operate in (e.g., near sandbanks), and perform actions to satisfy objectives, such as *survey* an area (for example with a sonar); and to *acquire* (the exact location of) a target. UAVs can have different configurations, which determine what tasks they can perform and how they behave when performing them. For example, a UAV with a camera can take underwater images. Figure 1 provides an overview of an inspection mission on a wind farm, including launch/recovery points, and survey areas (around certain wind turbines). The figure also plots the actions performed by the UAVs, including transiting, diving to the survey depth, and surveying an area using a lawnmower pattern behaviour.

We have modelled the scenario as a Temporal Planning Problem. The state of the scenario is represented using propositional and numeric fluents. We have used propositions to describe the structure of the problem, including the navigable connections between locations, the properties of each asset, and the properties of locations (e.g., that a location is part of a survey area). Propositions are also used to model the location of each asset. Numeric fluents are used to record the depth of the vehicles. We have implemented the main behaviours in the scenario as durative actions, including a move action, different survey actions, observation actions, and climb and dive actions. The duration of the actions is assigned based on an estimate of how long it is expected to take. For example, the move action duration is based on the distance between the start and end points, and the survey actions depend on their mode and the area to be surveyed. These PDDL models are generated directly from proprietary command and control end-to-end mission software.

We are developing a framework for supporting human operators throughout UAV missions. In this work, we focus on the problem of effectively supporting human operators as they plan UAV missions. To motivate this problem, we focus on four example objectives that an operator might be considering when observing a plan for these tasks:

- Objective 1: Due to the nature of the specific vehicles, the operator wants to reduce the distance travelled by one of the vehicles. For example, consider the case of an older vehicle, which has an older, and less reliable battery.
- Objective 2: Similarly, due to reliability issues (e.g., sensor issues) with a certain vehicle, the operator wants to manage the number of tasks assigned to that vehicle.
- Objective 3: The operator wants to reduce risk in the mission. For example, they do not want the vehicles to go close to exclusion zones, or go close to other vehicles.
- Objective 4: During a wind farm inspection mission, wind turbines can disrupt vehicle communication, and the operator is concerned with loss of communication.

**Fig. 2:**
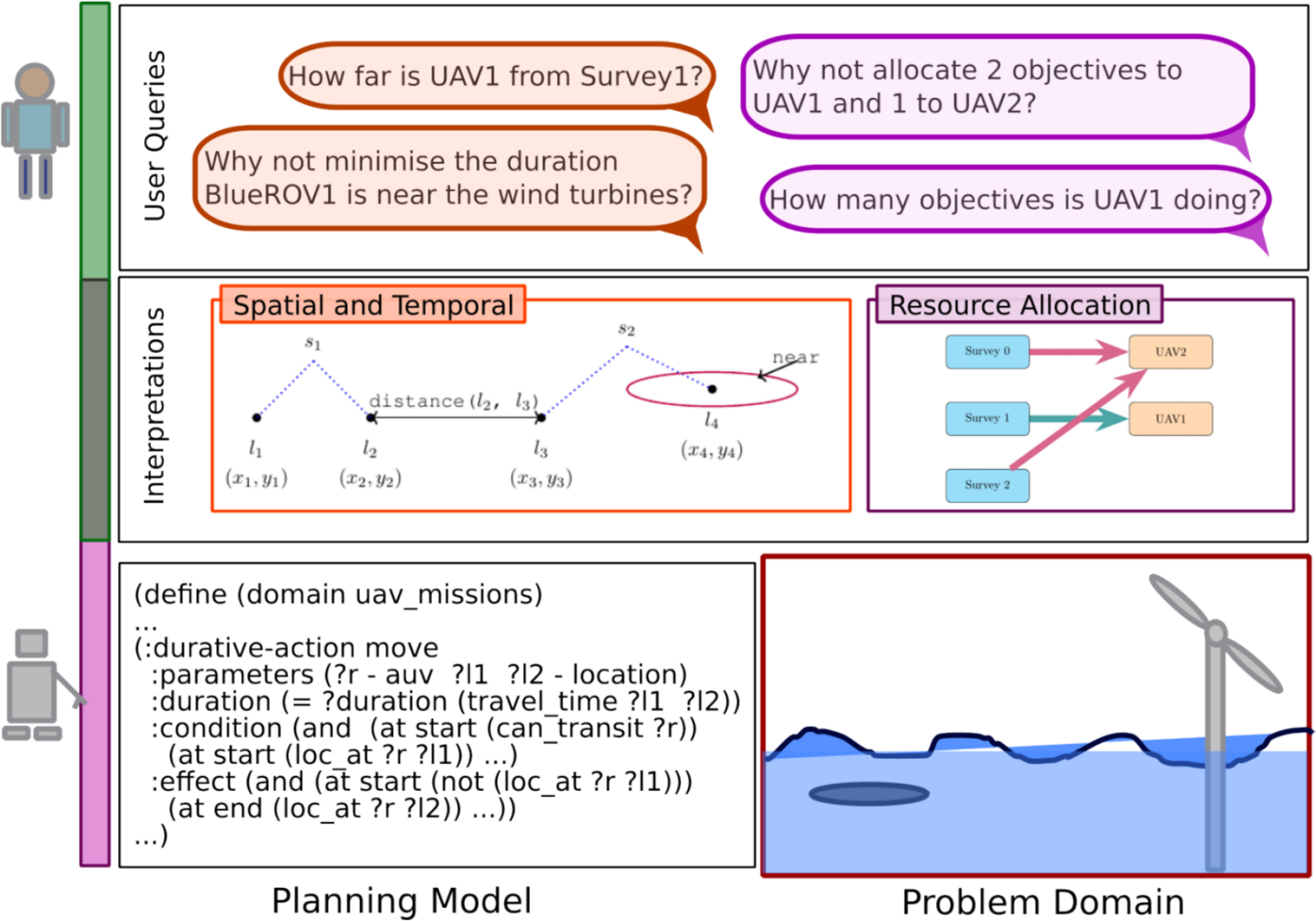
The planning model (bottom left) captures certain problem domain (bottom right) concepts necessary to support the plan language. The user may ask queries about the plan, which use additional concepts (top). We propose building a layer of model interpretations (middle), which support additional concepts from the problem domain, while also linking with, or extending the planning model.

## Human-agent representation misalignment

At a conceptual level we can consider a plan as a series of events, and a collection of features measuring various aspects over time. It has been common in XAIP to directly connect the events and measures available for user queries to those defined in the planning model, e.g., [7, 10]. The plan language associated with a particular planning model naturally describes certain events (plan actions) and measures (functions, propositions, or satisfied goals). The selection of this language is made during the modelling process. During the modelling process, the domain modeller must balance several factors, including conciseness, appropriate plan language (to allow the plan to be meaningfully executed), solvability, and readability. A main intention of the modelling process is to select a planning model that makes a concise description of the rules of the world [60]. In fact, a deliberate choice was made to separate out additional concepts, such as advice [60], and this has become common practice. This means that there are many events and measures that are deliberately missing from the planning model.

As a consequence, the user’s understanding of the problem domain and their interpretation of the plan will not always map directly onto the specific events and measures represented in the planning model. For example, when a user asks the query: *“What is the total distance travelled by the vehicles in this plan?”*, they are reasoning in terms of high-level, intuitive concepts such as *vehicles* and *distance travelled*. Even in this relatively simple case, misalignments can arise: the phrase *the vehicles* may need to be resolved to specific objects in the model (e.g., *BlueROV1* and *BlueROV2*); movement may be represented only indirectly through actions or facts rather than as an explicit distance property; and the necessary distance values might reside in external sources that must be integrated. This has been observed in [11] where they observe that the representation of a human user and the representation of the agent will typically not be the same. In particular, when the user interrogates the system, their descriptions of the problem and solution will likely include references to events and features that are not explicitly captured in the planning model. This is not surprising given the diversity of reference approaches that humans use during human-human interactions [61].

In this work we consider the question: *‘Does extending the concepts available for user queries allow for clearer communication with the system, and enable more useful explanations?’* We address this human–agent representation misalignment problem by introducing a middle layer that enriches the agent’s internal model with additional human-relevant concepts. This layer serves as a bridge between the formal structures used by the agent and conceptual structures aligned with human understanding, supporting more natural and accurate query interpretation.

Other strategies have been explored for bridging the representational gap. One approach is to attempt to align the agent’s internal representation with human conceptual structures, reducing the translation overhead [62]. Another is to map natural language queries to plan elements through semantic parsing or template matching [63]. A third strategy constrains the interaction schema itself, guiding the human to formulate queries that more closely match the agent’s representation, e.g.,  [7]. While effective in certain contexts, these methods can demand substantial domain re-engineering, large volumes of annotated training data, or impose limitations on user flexibility. Our middle-layer approach aims to retain the efficiency and scalability of the existing model while introducing an extendable collection of concepts, supporting richer, more intuitive interactions. Moreover, its use is not exclusive of these alternative approaches, but complementary. For example, in introducing additional concepts in a middle layer, we can reduce the query mapping problem.

We propose developing a middle layer. This layer extends the planning model with additional concepts that are relevant to the problem domain, but are not necessarily represented in the specific planning model. In particular, we propose extending the collection of events and measures that the user can refer to beyond those explicit in the planning model. We can think of the situation in three levels of concepts (see Fig. 2): those used in user queries, those supported in the planning model, and those defined our proposed layer of interpretations.

- **User Query:** Natural language queries which relate to the user’s representation of the plan, from their mental model of the system, their assessment of important measures of the plan, and their intentions for how it might change. Whereas the operator will have (deep) knowledge of the problem domain, they are unlikely to understand the specifics of the selected planning model. As such, their queries are likely to refer to aspect of the problem domain. The top of Fig. 2 presents example queries that an operator might ask in the UAV scenario. These include a question about the total distance travelled by a vehicle, and a query about the number of tasks that an asset is performing.
- **Interpretations:** Components that provide interpretations of the planning model, and support additional concepts combined with automatic model extensions (see the middle of Fig. 2). In this work we consider spatial-temporal and resource allocation interpretations, which provide concepts such as distance, and the allocation of resources to tasks. These additional concepts can be used in user queries. Moreover the concepts are connected to the structure of the planning model, so that the concepts can provide new ways to interrogate and manipulate the planning model.
- **PDDL Model:** The representation used by the system to generate plans (see the bottom left of Fig. 2). The planning model identifies specific robot behaviours, such as the robot moving between waypoints, altering its depth, and the robot surveying an area, or observing a specific point. The model is appropriate for capturing concisely an appropriate state transition system for the scenario, and as noted in the text: the domain modellers must balance several factors when designing a domain model (e.g., conciseness, appropriate plan language, solvability, readability).

The intention is that introducing appropriate concepts can help to bridge the gap between the human and agent representations. These concepts extend the underlying planning model with well defined concepts, which can provide new views and interpretations on plans and the planning model, and also allow new ways of modifying and extending the planning model. Notice through building these concepts from the underlying planning model we can be precise about the interpretation of concepts (e.g., distance). This layer then provides additional concepts that can be mapped to in user queries, supporting additional user queries and extra interpretation in system responses.

### A structure dependent concept layer

In this work we explore bridging the human-agent representation gap. Our approach aims to establish a middle layer, sitting between the domain specific concepts, and domain independent concepts (e.g., actions and functions), which supports additional concepts for use in human-agent interaction. These concepts are organised around specific model structures (e.g., navigation). The motivation for this is that planning problems often share structures, such as navigation, transportation, manipulation, and resource management. Through developing concepts around these structures we can define the concepts once, and make them available for any domain with the structure. Moreover, as we are considering smaller structures of the model, it is possible to consider specific concepts relevant to the structure. Motivated by our UAV case study, we have focused on spatial-temporal and resource allocations interpretations of the problem. In the following sections we consider these interpretations as part of the multi-agent spatial-temporal (MAST) structure, and demonstrate how it can support additional concepts.

## Multi-agent spatial-temporal (MAST) structure

In this work, we have identified that many of the types of interesting queries in our scenario relate to multi-agent, spatial and temporal relationships. In this section, we define a multi-agent spatial and temporal (MAST) structure, which is a substructure that is part of many common planning problems. This is similar to a *generic type*, which is a common structure that exists across planning models [64]. The intention is to establish a middle abstraction layer, which can allow concepts to be shared between different problems, while also providing a suitable level for the supporting knowledge to be expressed concisely and effectively.

The MAST definition captures the connection between the planning model and the MAST concepts (navigation, resources etc.). Notice that although the MAST definition is in terms of a specific planning model, the connection with the MAST concepts is made once for a planning domain (see Subsect. 5.2). As well as this, the MAST structure also extends the navigation information content defined in the temporal planning model with scenario specific data (locations, collections of locations etc.), which are not typically part of the planning model. We first present the MAST definition and then present our specification language for MAST structures, which allows the user to identify the relevant elements of a temporal planning model, and to provide the necessary additional scenario information.

### The MAST structure

#### Definition 2

We define the multi-agent spatial-temporal (MAST) structure: MAST = $\langle P, \mathbb {N}, \mathbb {T}, \mathbb {L}, \mathbb {S} \rangle$, such that *P* is a temporal planning model; $\mathbb {N}$ is a set of navigating objects of the form $\langle n, \delta \rangle$, where $n\subseteq O$, and $\delta$ is a movement model; $\mathbb {T}=\langle label, \theta \rangle$ is a set of task descriptions, with label, and satisfaction expression ($\theta$); $\mathbb {L}$ is a set of discrete locations of the form $\langle l, coord\rangle$, where $l\in O$ and *coord* is a point coordinate; and $\mathbb {S}$ is a set of labelled structures (e.g., a specific wind turbine, or the collection of vehicles).

The definition relies on establishing a connection between the MAST structure and the underlying planning model. This is essential to support the MAST interpretations to automatically extend the planning model appropriately with additional structures (e.g., a new numeric functions monitoring distance travelled).

*The Movement Model* The movement model, $\delta$, for a navigating object, *n*, defines the structure in the planning model that supports the navigation behaviour of *n*. The model identifies the location predicate (e.g., loc_at ?n ?l), and navigation actions (e.g., move). In our UAV scenario, the move action is the navigation action. The objects that move are the vehicles, and the loc_at predicate determines their current positions. The specific descriptions of these structures are presented in ‘Spatial-Temporal Interpretation’ below.

**Fig. 3:**
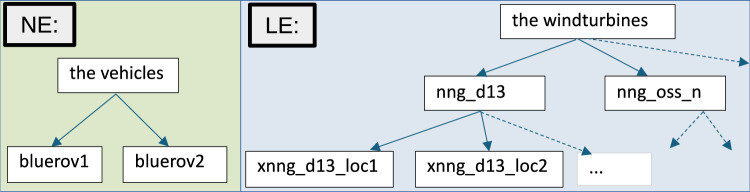
Objects in MAST problems can be naturally gathered into hierarchies, with appropriate labelling at each level of the hierarchy.

*Task Satisfaction Expression* In this work we have adopted a basic task satisfaction expression (TSE) to identify the completion of a task (see e.g.,  [65, 66] for approaches for extending this approach). In particular, each task is associated with an action name, and a set of annotated parameters. The annotations are drawn from the set: ${\_,ID0,..,IDm,MOVER}$, where $`\_'$ is unspecified, *MOVER*, is assigned the navigator asset, and the tuple (*ID*0, ..., *IDm*) with the action name identify the specific task. For example, in our UAV scenario, the tasks are the observation of targets, and the surveying of survey areas. In each case one of the UAVs performs the task and can be identified in the action arguments. The tasks are identified by either the survey area object, or the location object, depending on the task type. The specific descriptions of these structures are presented in ‘Resource Interpretation’ below.

*Labelled Structures* The definition of labelled structures allows us to build up a collection of location and navigating entities (denoted *LE* and *NE*). For example, Fig. 3 illustrates part of the hierarchy of structures for our scenario. On the left side, the navigating entities are presented: each of the individual vehicles, and the collection of all vehicles. The navigating entities in this scenario are: ‘BlueROV1’, ‘BlueROV2’, and ‘the vehicles’. On the right of the figure, the location entities are presented. This includes each of the locations, including launch, recovery or other landmarks, and each location of a survey area, a wind turbine, or an exclusion zone. There are then labelled entities for each of the wind turbines (e.g., wind turbines ‘NNG_D13’ or ‘NNG_OSS_N’), which is made up of the points that outline its shape in the planning model; at the top, a single entity includes all wind turbines. There are also similar entities added for the other areas (e.g., survey areas and exclusion zones). Notice that the mission planning software that we are using represents these structures, allowing us to populate this information directly.

**Fig. 4:**
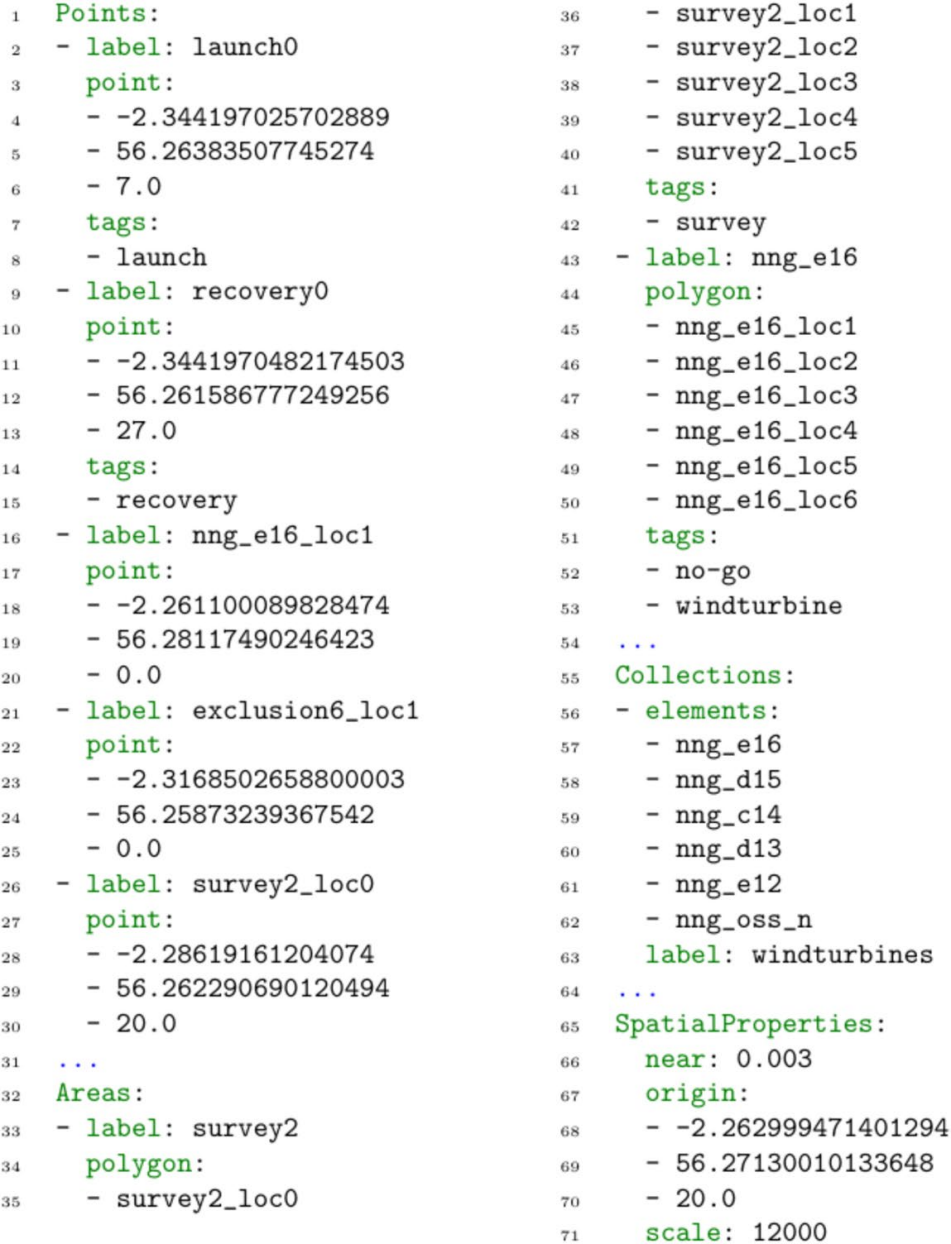
An example of the specification of scenario spatial concepts in the YAML language. The specification connects the PDDL location objects with their real-world coordinates (the Points list), defines polygons from groups of these objects (the Areas list), and defines collections of points and polygons (the Collections list). There is optional tagging for these structures, and also some additional spatial properties.

### MAST specification

The connection between the concepts and the planning domain has two parts. The first is the domain connection, which requires PDDL elements to be associated with the structure’s concepts. In order that the concepts can be appropriately interpreted in the original planning model it is necessary that there is a clear mapping between the concept and the planning model. This is essential here to allow the planning model to be appropriately manipulated. This is done once for each domain.

The second part is the scenario data. This information (e.g., the coordinates of locations, and naming hierarchies) is not typically defined in the planning model. However, in many real world applications this form of data is readily available.

The specification of a MAST problem in our system is made with the tuple $\langle$ *P*, *DomainInterpretation*, *ScenarioData*$\rangle$, with *P*, a temporal planning problem (in PDDL), *DomainInterpretation*, a specification between the problem domain structure and interpretations (e.g., visualisation, and MAST concepts), and *ScenarioData*, which provides the problem specific data, including specific point information. Here we present the representation language for specifying the scenario and domain information.

#### Scenario data

The main aim of the scenario data is to define the spatial data for the scenario, including the coordinates, shapes, and connected groupings. Firstly, the inclusion of coordinate information allows the system to support distance based concepts, and spatial analysis (e.g., total distance travelled, or determining how close a moving object got to a certain point). This is supplemented by area and collection information, which establish collections of points, associated labelling, and their appropriate interpretations. It starts with a set of PDDL locations with their associated coordinates. These are then grouped into areas, and collections. Each area has a name and is described by a sequence of points (a polygon). Each collection gathers points and polygons. Figure 4 presents an extract from a YAML specification for a UAV scenario.

*Points* The list of points (lines 1–27) connects the PDDL location objects with (potentially) real world coordinates. Each point defines the location object (label), and the coordinate (point). Points can also have optional tags (e.g., to identify target objectives). For example, lines 2–8 describe an asset’s launch position, with the name *launch0*, coordinates $(-2.344197025702889, 56.26383507745274, 7.0)$, and a launch tag.

*Areas* The list of areas (lines 28–50) indicate that some of the locations are part of connected shapes. For example, the exclusion zones and survey areas are both defined by a set of points describing their boundary. These shapes are described by a name (label), and an ordered list of location objects (polygon). Areas can also be tagged. For example, in lines 33–42, the area *survey2* is defined as the connected polygon, with points *survey2_loc0*,..,*survey2_loc5*, and tagged as a survey.

*Collections* The list of collections (lines 51–60) indicates collections of spatial structures (points and areas), that are also related. For example, we can bring together the set of all wind turbines, survey areas, and exclusion zones. For example, in lines 56–63, the *windturbines* collection is defined, as the collection of individual wind turbines: *nng_e16*, *nng_d15*, ... (which are each areas).

*Additional Information* The language also supports some additional scenario specific data to be provided (lines 61–67). In particular, values for the scale, a reference point for the scenario (the origin), and the appropriate interpretation of the concept near can be provided.

**Fig. 5:**
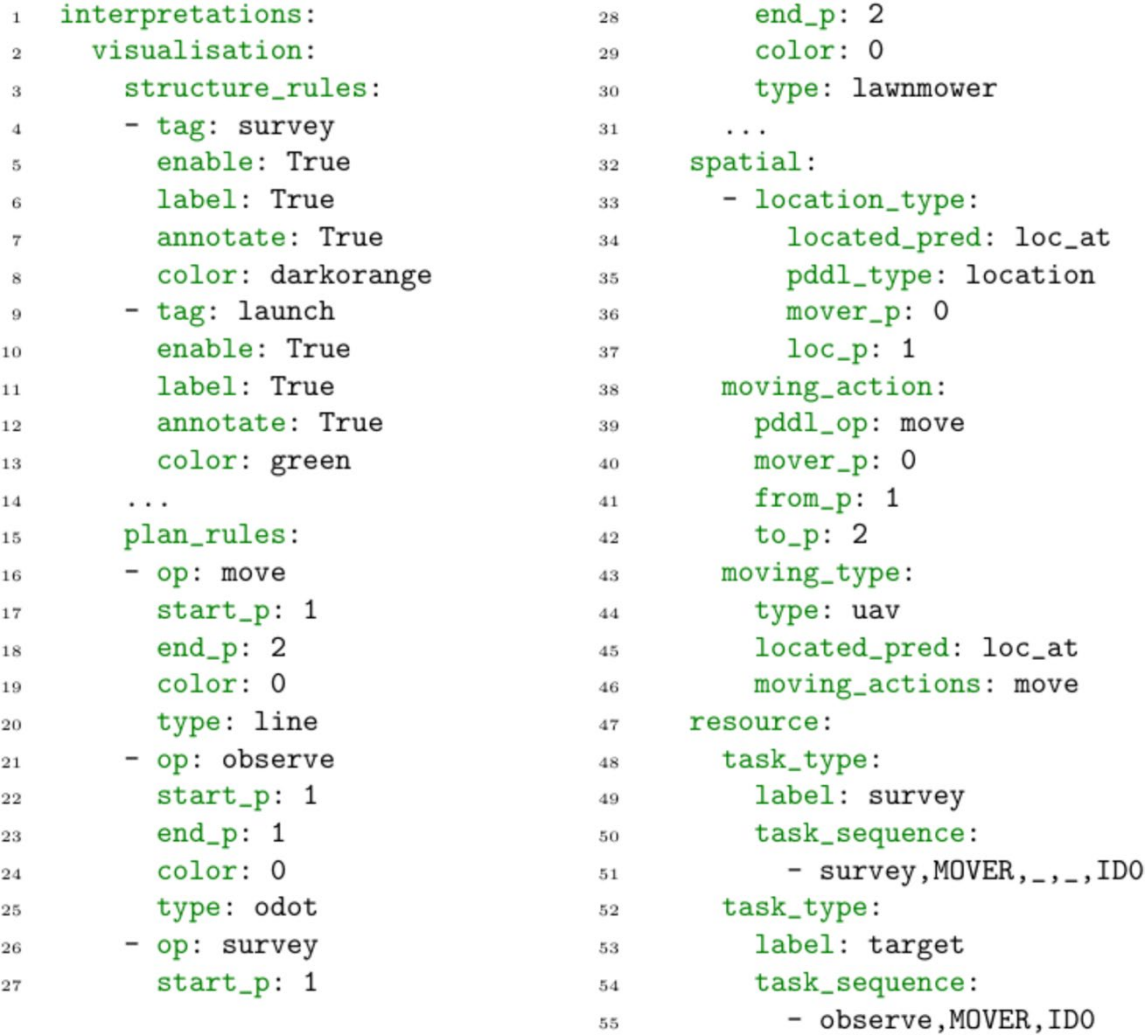
An example of the specification of the domain interpretation in the YAML language. The specification connects the PDDL structure with MAST concepts (e.g., movers, and tasks). There are several interpretations. The visualisation interpretation specifies the connection between the spatial elements, the actions, and the visualisation concepts. There are also interpretations for establishing the connection between the planning model, and the MAST concepts (spatial and resource allocation).

#### Domain interpretation

The domain interpretation provides a mapping between the model structure, the scenario data, and MAST concepts (including the visualisation). The interpretations include visualisation, spatial-temporal, and resource. The visualisation interpretation requires information about how the scenario locations should be drawn, and how actions should be plotted. This provides the rules necessary for the system to build plan and plan comparison visualisations, allowing the system to provide visual feedback during the interaction. The spatial and temporal interpretation requires details about the move actions, the objects that move, and the locations they move between. These details allow the interpretation to appropriately interpret and manipulate the model (e.g., monitor the distance travelled by a moving object). The resource management interpretation requires details about the problem’s tasks, and the relevant resources. This allows the system to analyse plans, or manipulate the model (e.g., control the tasks serviced by a resource). Figure 5 presents an extract from a YAML specification for our UAV domain, including the system’s parameterisation for the visualisation, the spatial and temporal, and resource allocation interpretations.

**Fig. 6:**
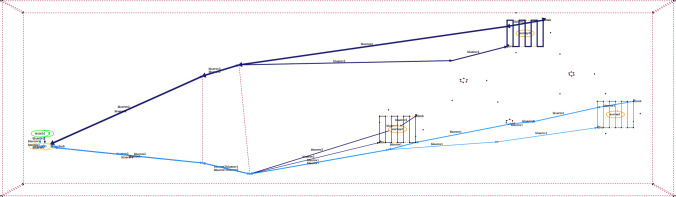
The figure presents a visualisation of a plan with two assets. The actions are plotted with a light blue line for BlueROV1, and dark blue for BlueROV2. On the left are the launch and recovery. There are exclusion zones (red dotted lines), three survey areas, and six wind turbines (including in the centre of the survey areas).

**Fig. 7:**
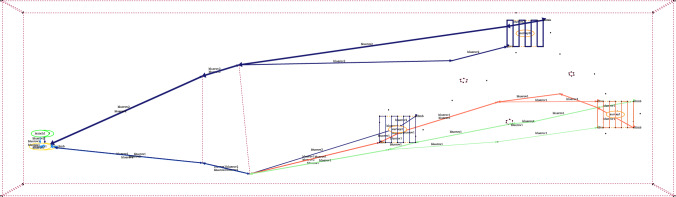
The figure presents a visualisation to illustrate the comparison between two plans. The blue lines indicate parts of the plan that are the same in both plans; the green lines are from the original plan; and orange/red lines indicate actions in the new plan.

*Visualisation Interpretation* In our UAV scenario, the operators are familiar with plan visualisations, and we have developed a similar graph based visual language, in order to support effective communication of plans and plan comparisons. The plans are plotted over a top-down view, identifying the mission’s key points, and structures. The approach provides a general approach to generate graph based visualisations from plans.

**Specification** The visualisation interpretation (lines 2–19) provides the rules to create a visualisation based on the spatial structures, and the plan actions. It has main two parts: the structure rules, which indicate any special rules for drawing the scenario (e.g., using a specific colour to indicate no-go zones), and the plan rules, which describe how each action will be visualised.

The structure rules allow for the important structures of the scenario to be visualised. The rules are based on the tagging of location entities (see Fig. 4). For example, in the excerpt above, the polygon representing a survey area 2 was tagged with survey. In line 21 of Fig. 5 the rule indicates the visualisation rule for this area. It indicates that the area should be drawn (line 22), that it should have a label and an annotation (lines 23–24), and that its colour should be dark orange. There are default tags (point and area) that can be used to provide default behaviours.

The plan rules indicate how each of the plan actions should be visualised. Each rule indicates the planning action, the type of visualisation, the start and end locations for the action, and the colour. The basic visualisation is a line, which naturally represents movement. We also support other types, including customised visualisations. For example, the survey action represents an asset performing a series of move behaviours in a lawnmower pattern. We therefore have created a specialised visualisation for lawnmower pattern. In particular, given a survey area (a polygon), we generate a set of points and edges that create a connected lawnmower pattern over the survey area. The line thickness for any action depends on the time of the action. In particular, it is determined by the action’s midpoint (start time plus half its duration). The thickness is scaled between a minimum and maximum thickness. The intention is that this makes it easier for the user to understand the flow of the plan.

**Plan Visualisation** Figure 6 presents an example plan visualisation for the UAV scenario. The structure of the scenario is plotted using the points and areas from the scenario data, using the rendering rules provided in the structure rules. There are launch and recovery waypoints for the two assets (BlueROV1 and BlueROV2), a sandbank, six wind turbines, and three survey areas. In this scenario the goal is to survey the bases of three of the six wind turbines, and return the assets to their recovery points. The plan actions are then added, with different colours selected for each unique object identified by the plan rules. In the figure, the actions are plotted with a light blue line for BlueROV1, and dark blue for BlueROV2. In this example BlueROV1 peforms survey 1 (far right), and BlueROV2 performs the other two surveys (survey 0 at the top, and survey 2 in the middle). For each survey the asset moves to the survey area and dives to the correct survey depth. It then performs a lawnmower pattern survey, and climbs back to transit depth.

**Plan Comparison Visualisation** To assist the operator in comparing the plan pairs ($\pi _i$, $\pi _{i+1}$), we have implemented a plan comparison visualisation. As with existing work in XAIP we have used different colour maps in order to emphasise the comparison [7, 22]. Figure 7 presents an example plan visualisation for the uav scenario. For the original plan ($\pi _0$) and the new plan ($\pi _1$), we generate three line segments: the parts where the plans match (presented in blue); the part of the original plan $\pi _0$ that is not in the new plan $\pi _1$ (in green), and the part of the new plan $\pi _1$, which is not in the old plan (in red/orange). To do this, we used the Levenshtein distance [67]: the distance between two-word sequences, which provides a measure of the edit difference between the sequences while also respecting order. In our case, we use unique words for each ground action and extract the best match between the two action sequences. In Fig. 7, the change leads to BlueROV1 taking a higher path on its way to and from Survey 1 (orange), whereas in the original plan, the path was more direct past wind turbine (nng_d13). In the comparison, the part of the plan involving BlueROV2 (dark blue), is the same in each plan.

*Spatial-Temporal Interpretation* The spatial-temporal interpretation provides the connection between the model structure and MAST spatial-temporal concepts. The specification identifies the relevant actions, predicates, and parameters to determine the location type, the moving actions, and the moving types. For example, the specification in Fig. 5 for the uav scenario in lines 33–43, specifies that the moving type uav (in the PDDL model), exchanges located predicates (loc_at), using move actions. It also details the important parameters and types for the action (moving_action) and predicate (located_pred).

*Resource Interpretation* The resource interpretation provides the connection between the model structure and the MAST resource allocation concepts. It has been assumed that the set of moving entities are the assets. This assumption simplifies the specification, and is common to many navigation tasks (including our UAV scenario). However, it is not an important assumption for the concepts in the paper. The specification involves a list of task types, such as surveys and targets. These task types can be used in order to identify a set of ground tasks in the specific scenario. The tasks are defined by a name, and an action header. The action header indicates several key features for the task. In particular, they specify the asset, and an id, which distinguishes the task. In the specification excerpt, there are two task types (lines 45–52): surveys and targets. The action header for the survey task (line 48), indicates that unique tasks are identified by the fourth parameter, and that the asset is the first parameter. A similar template is used for the observe action. For each scenario, the set of tasks is generated using the id elements, and the reachable action set.

The MAST specifications for an instance of the rovers and driverlog benchmark domains are presented in Appendix A.

**Fig. 8:**
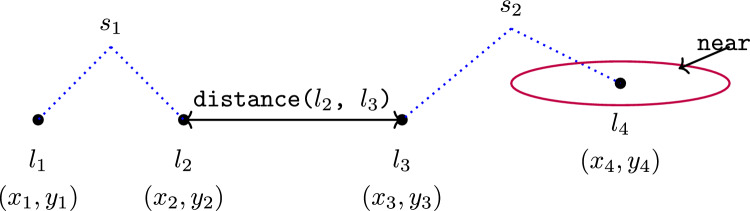
The MAST structure defines locations, their coordinates, and structures over these points. Relationships, e.g., the distance between points, can be defined over this structure.

## Extending the model with MAST concepts

Many XAIP approaches assume that an operator’s questions correspond directly to concepts in the planning model. However, as illustrated by the example operator objectives in our scenario (see Subsect. 3.3), there is not always a direct mapping between user-level concepts and those represented in the model. These example objectives were suggested by our industry partner to demonstrate the kinds of knowledge operators typically use but which are not captured in the planning model (e.g., that an asset has a weak battery, or that maintenance is scheduled in a particular area). These objectives motivated the selection of the concepts explored in this work. We note that our approach does not aim to guarantee complete coverage of all relevant concepts; rather, it demonstrates (i) how such concepts can be added to support effective communication, and (ii) how these concepts can potentially be shared across domains. A complementary approach could involve observing or interviewing users as they interact with plans to identify additional important concepts, an idea we consider further in Sect. 9. In this work, we employ our MAST structure to support the extension of the planning model with these additional concepts.

### The MAST spatial and temporal concept wrappers

We adopt a framework where a layer of interpretation is built on top of the MAST structure, providing basic spatial and temporal concepts and relationships. For example, Fig. 8 presents some of the functions that can be supported by the MAST structure. The distance relationship can be calculated directly for pairs of location entities, using the length between the two entities. We have used this framework in our system and these relationships to support a collection of concepts, relevant to our autonomous vehicle scenario. Each concept is paired with a procedure to add its corresponding structure to the problem model.

**Fig. 9:**
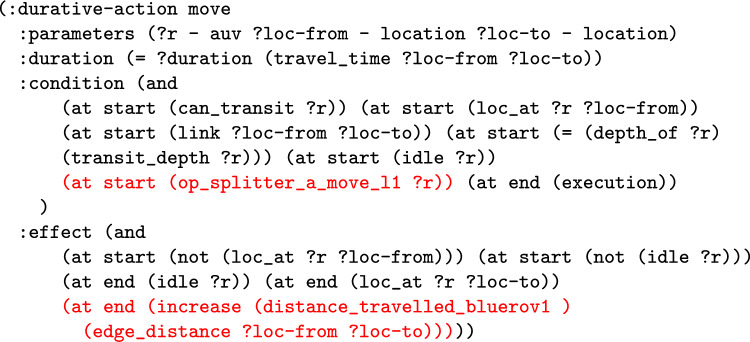
The split move action for BlueROV1, which will track the distance it travels (added structure in red).

**Fig. 10:**
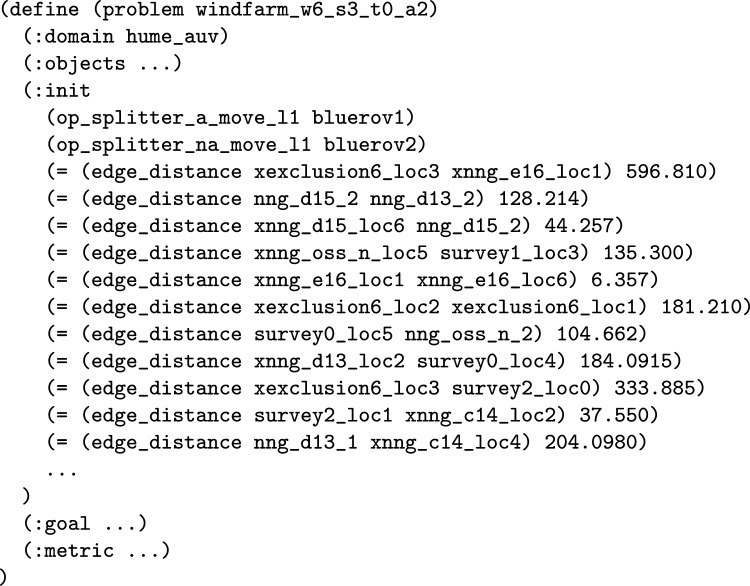
Part of the problem file for the extended UAV scenario, with distances between edges (calculated from the MAST data), and special propositions used to split the move operator.

**Fig. 11:**
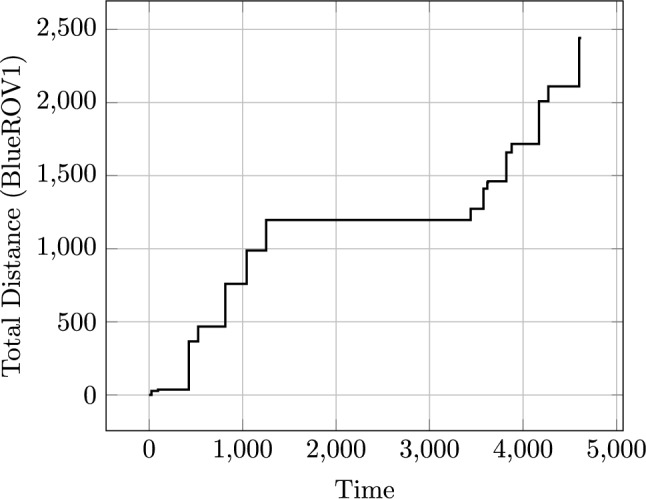
A plot illustrating the value of the new function monitoring the total distance travelled for BlueROV1, during a plan.

*The Total Distance Navigated by a Navigator* The first wrapper adds a new numeric fluent to the planning model called distance_travelled_by_*NE*, for a navigating entity. The intention is that the function will accumulate the distance travelled by the particular navigating entity. The first step is to extend the model with the data required. The model is extended with the edge_distance function, which has two location parameters, and records the inter-location distance in its parameters. The function then automatically adds structure to the move actions to record the distance travelled by each of the move actions of the navigating entity. Notice that the entity could be an individual navigating object, in which case the planning operator is duplicated. One version is used for *NE*, and the distance function is used to increase the distance travelled function. The other version is used for the rest of the vehicles. In the case that *NE* includes all the vehicles, the move action does not need to split, and an increase effect is implemented for all vehicles.

**Example:** Distance travelled functions can be added into the model to monitor the distance travelled by a navigation entity, or entities. For example, in our UAV scenario, we can consider adding a monitor of the distance travelled by BlueROV1. In consequence, the system will extend the model with a new function, that will be used to accumulate the distance travelled by BlueROV1. This requires that the relevant moving planning operators are split (e.g., the *move* action), so that actions related to BlueROV1 are separated from other vehicles. The operator associated with BlueROV1 has an additional effect added in order to monitor the distance travelled. Figure 9 presents the PDDL of the operator for BlueROV1. The initial state (see Fig. 10) is then extended with distance propositions, and propositions to enforce the appropriate application of the split operators.

We can monitor this function as it accumulates during the execution of a plan. Figure 11 plots the distance travelled by BlueROV1 against the time in the plan.

**Fig. 12:**
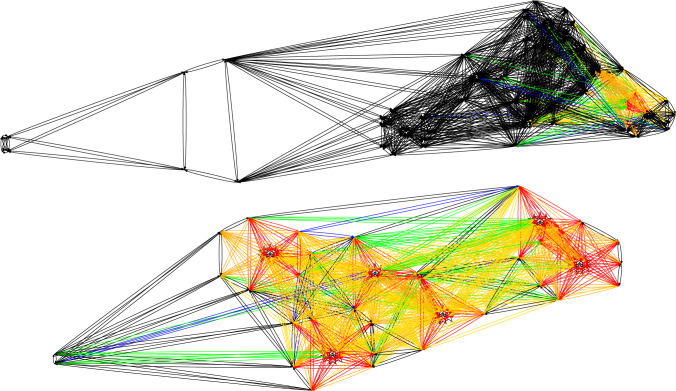
The figure plots the proportion of each navigation action that is near to one of the located entities. In the top figure, the entity is one point on Survey1, and in the bottom figure the entities are all of the windturbines. The top figure is generated for our simulated scenario (windfarm) used in the user study, and the bottom figure is generated for a mission that was tested in a real quarry (quarry). In the figures, the proportion of the line near to the point is mapped to a colour using the following rules: >0.7:red, >0.4:orange, >0.2:gold, >0.1:green, >0:blue, =0:black.

*The Duration that a Navigator is near a Location* This wrapper adds a numeric fluent to the planning model called duration_*NE*_near_*LE*, for a navigating entity (*NE*) and location entity (*LE*). The intention is that the function will accumulate the duration that the navigating entity is near to the location entity. The first step requires that each edge is analysed to determine whether they are near to the location entity. The function identifies the points and areas that are part of the location entity. It analyses each edge, identifying the proportion of the edge that is near to an entity. This can be achieved by first splitting the edge at points that cross between near and not near for any object, labelling each segment as near or not near, and finally calculating the proportion. This is repeated for each edge and used to populate a new (static) numeric fluent in the model (proportion_edge_near_*LE* ?x ?y - location). A similar procedure can be used to identify if the individual points are near the entities. Figure 12 presents proximity plots, which depict the proportion_edge_near_*LE* function for the transit paths that are near one point of a survey.

**Fig. 13:**
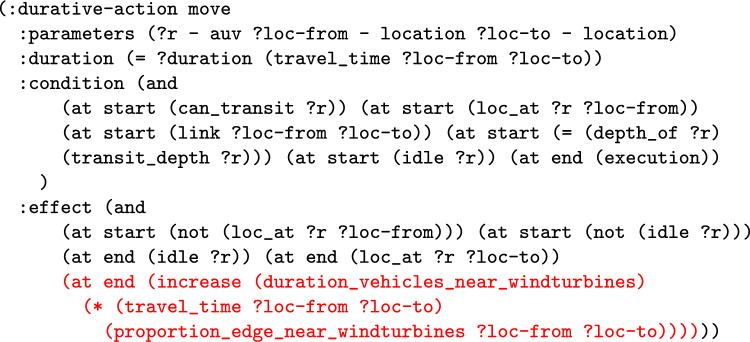
The updated move action for all vehicles, which will track the duration that a vehicle is near to a wind turbine (added structure in red)..

**Fig. 14:**
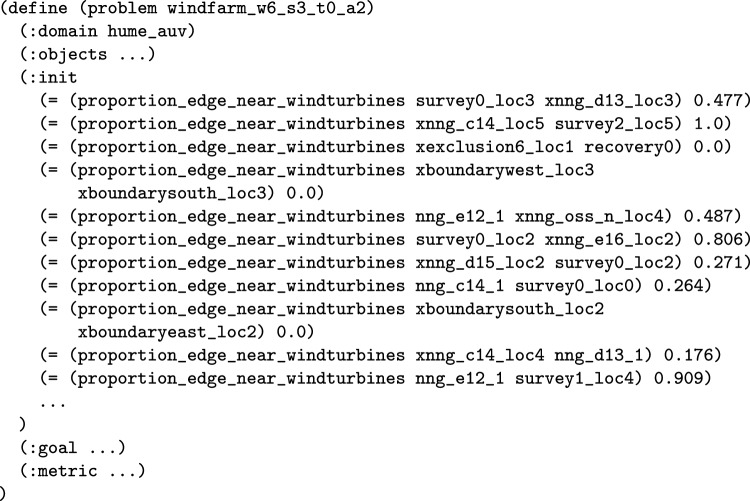
Part of the problem file for the extended UAV scenario, with functions recording what proportion of each edge is near to a wind turbine (calculated from the MAST data).

A function is then added to the model to accumulate the specific duration being monitored, e.g., (duration_*NE*_near_*LE*). The wrapper identifies the relevant move actions (using the move action model) and these actions are then updated to accumulate the appropriate duration. The movement action is updated by multiplying the duration of the move action by the proportion of the edge near the entity. Although our implementation assumes a linear model of movement, the approach is general, and as these values can be preprocessed, any model can be used.

The appropriate behaviour for other actions can be customised to accumulate time appropriately for the problem. In actions that are conditioned on the located predicate, we can use a similar process to extend these actions with duration accumulating effects, but in this case based on whether the specific point is near. In the case that the actions of navigators interact with time-dependent actions (either other navigators or timed initial literals), a navigator might have to wait. So that the model ensures that all periods are considered we can extend the model with an idle action, with the appropriate clip-actions [68], to ensure that the time of each navigator is recorded. Although our system supports this functionality, we have found that it adds complexity and is often not required in practice.

It is worth noting that these language extension modules also register new functions with the natural language processing part of our system (see Sect. 8), and provide a set of examples to support it in parsing queries.

**Fig. 15:**
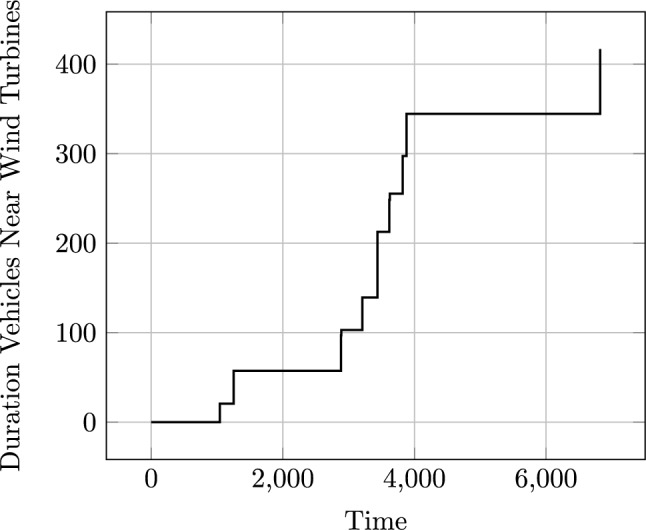
A plot illustrating the value of a new function monitoring the total duration that the vehicles are near to the wind turbines, during a plan.

**Example:** Duration near functions can be added into the model to monitor the duration that a navigation entity is near to a location entity, or entities. For example, in our UAV scenario, we can consider adding a monitor of the duration that a vehicle is near to the wind turbines. In consequence, the system will extend the model with a new function, that will be used to accumulate this duration. This also requires that the relevant moving planning operators are extended with an additional effect to make the appropriate increment. Figure 13 presents the extended PDDL. Notice, because the query covers all of the navigation entities, there is no need to split the move operators. The initial state (see Fig. 14) is then extended with proportion propositions, which record the proportion of each edge that is near to the wind turbines.

We can monitor this function as it accumulates during the execution of a plan. Figure 15 plots the accumulation of duration as the vehicles move passed the wind turbines during the plan.

**Table 1:**
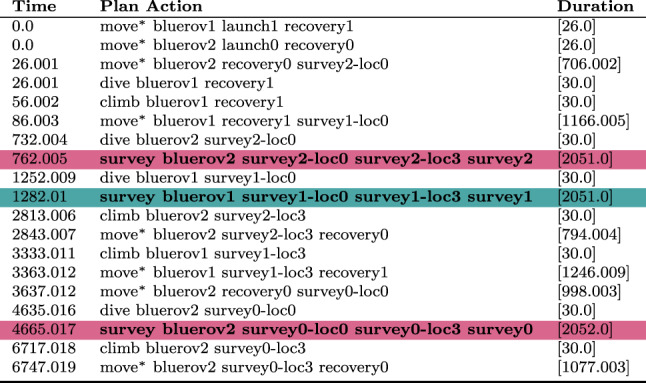
The initial generated plan with resource allocation (see Fig. 16a ) actions emphasised (purple for BlueROV2 and teal for BlueROV1).

Move actions have been collated where appropriate

**Fig. 16:**
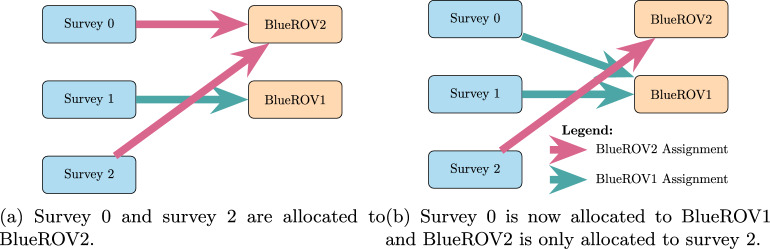
Allocation of assets to tasks before **a** and after **b** a resource allocation user query.

**Table 2:**
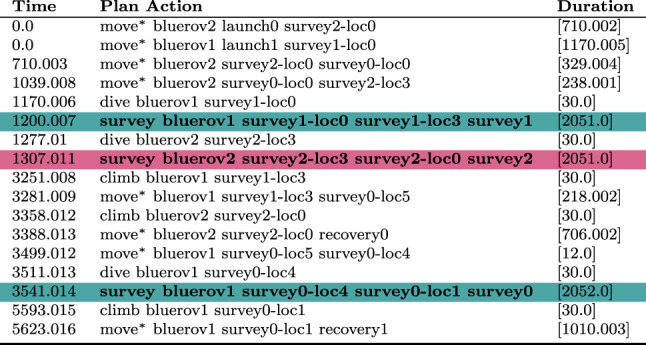
The generated plan after the user query, with resource allocation (see Fig. 16b ) actions emphasised (purple for BlueROV2 and teal for BlueROV1).

Move actions have been collated where appropriate

### The MAST resource management concept wrappers

A key aspect of the UAV missions is the allocation of the assets to different tasks. This is reflected in the existing interface of the proprietry software, where the allocation plays a central role in the operator’s interaction 69. In this section we identify a constraint for enforcing a specific resource allocation.

*Manipulating Resource Allocation*

We support functions based on resource management. The first of these identifies the allocation of an existing plan. In particular, given a plan, the function returns a mapping from tasks to assets (in this case navigators), $RA(\pi ):\mathbb {T}\mapsto \mathbb {N}$. This function evaluates the task satisfaction expressions on the provided plan. The expressions are defined to determine both the task, and the associated asset. The function therefore processes the plan and builds a dictionary that identifies the allocation implicit in the plan.

*Monitor N* *to* *T* *Allocation*

The second function adds the structure to the model to monitor a certain allocation of a navigator to a task. This function translates the task satisfaction expression into a series of model updates. In practice this is similar to the constraints added to ensure an action is applied in [7]. In particular, predicates with prefix progress_marker are added to monitor the progress of each task, as it is satisfied. As with the monitoring functions above, the operators are split where appropriate using predicates with prefix op_splitter.

**Fig. 17:**
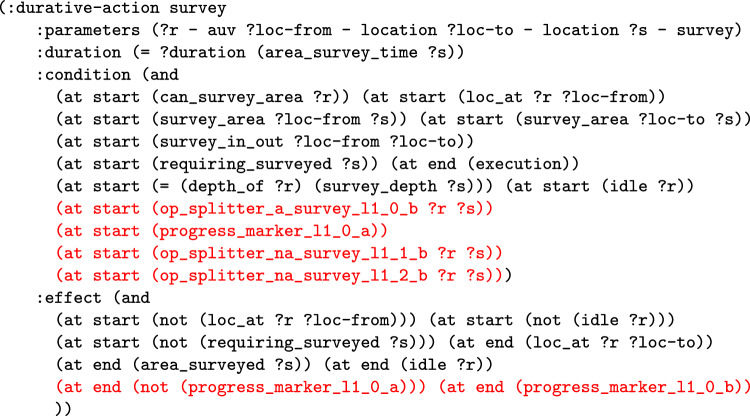
PDDL representation of one of the updated survey actions, which records the satisfaction of one of the tasks (added structure in red).

**Fig. 18:**
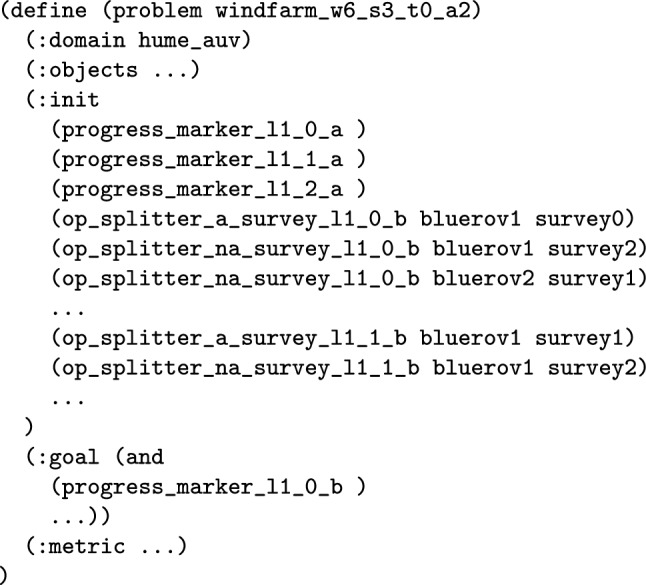
Part of the problem file for the extended UAV scenario, with propositions recording the progress of task allocations, and propositions to appropriately split planning operators. Progress markers are also added to the goal to ensure the tasks are satisfied by the intended assets.

*Force Allocation of N* *to* *T*

This function allows an (partial) allocation to be forced. In particular, a set of allocations can be specified. The function uses the monitor function to first add the structure to monitor each of the allocations. Then it adds a goal using the appropriate progress_marker predicates.

**Example:** Resource allocation queries can be used by the user to query why the system has not made specific (partial) resource allocations. For example, in our UAV scenario, the initial plan (see Table 1) might allocate surveys 0 and 2 to BlueROV2 and survey 1 to BlueROV1 (see Fig. 16a ). If the user then asks the query *‘Why not make the allocation of Survey0, and Survey1 to BlueROV1 and survey2 to BlueROV2 in the plan?’* then the system will add the necessary constraints to enforce this allocation and create a new plan (see Table 2). Figure 17 presents one of the extended survey actions. The action records the progress for one particular task. The corresponding problem file fragment is shown in Fig. 18. The progress_marker_X_a and progress_marker_X_b predicates record progress for each task. The op_splitter_a_X predicates determine the allocations that must be tracked. In the case, the survey action is applicable in situations where BlueROV1 is performing survey0. The goal ensures that this has been completed. If we analyse the new plan (see Fig. 16b ) we will see the new allocation.

### The MAST based model abstraction

In defining the MAST interpretations we have examined alternative views of the problem domain, at a higher level of abstraction. Of course, the user queries might actually relate to a lower level of abstraction than is an appropriate level of representation for solving the problem. One way of framing this is to model the problem at a low level, allowing queries to be mapped onto the structure, and then using an abstracted model during planning, ensuring the user’s query is appropriately represented in the abstraction. In order to explore this idea, we present an abstraction based on the abstraction in [70]. The abstraction is appropriate for MAST problems with restricted movement actions, where the actions have static cost for navigation. The approach also allows abstracting locations from the problem, by identifying a subset of locations where decision making is necessary.

**Fig. 19:**
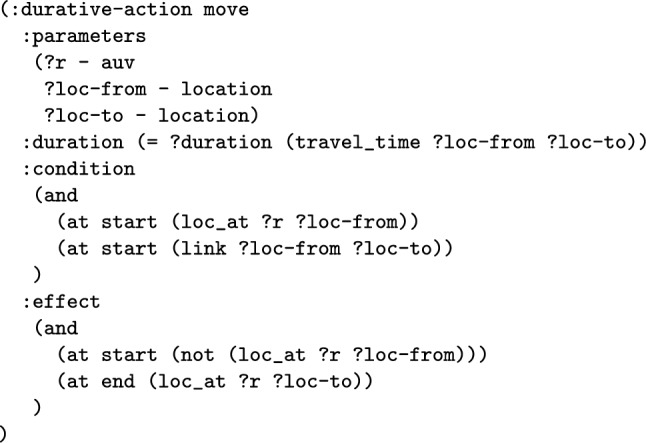
The PDDL representation of a basic move action.

**Fig. 20:**
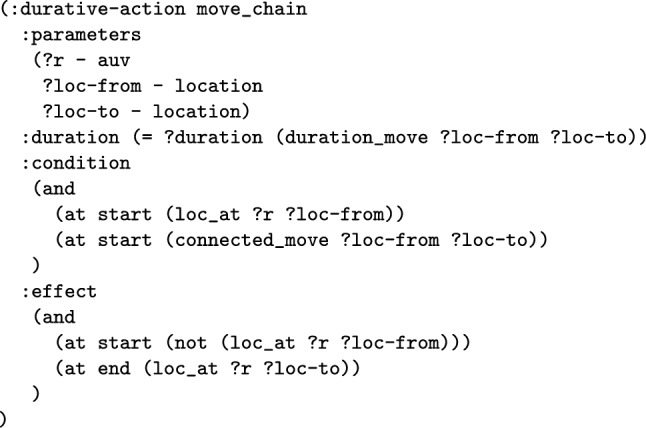
The PDDL representation of the composed move action.

*The Move Macro* Move actions in MAST structures are characterised by sets of *locations* and *movers*, a located predicate (e.g., at), which determines a one to one relationship between movers and locations. Consider the basic move action in Fig. 19. The action transfers the located relationship from the starting location to the final location. There is a static constraint (link), which is a function from movers and location pairs, which determines whether a mover can transition between two locations. There is also a function (travel_time), which specifies the duration of the action for the specific parameters.

The first step in the abstraction is to replace the navigation actions with macros that represent sequences of the navigation actions. This process must determine the appropriate conditions, effects and duration for the new macro action. We assume in this work that the specific conditions on the pairs of locations that can be traversed between is static (for a more detailed description see static graph in [64]), and that the duration, and numeric effect of the action are also static (they do not depend on the current state). We can therefore precompute these aspects of the action. This is done by extracting the connection map from the original model, and annotating each edge with both its duration, and its effect on each numeric variable. If we consider the map’s node set as *MapNodes*, we then can generate shortest paths for each of the pairs of nodes in the map. A connected proposition can be defined for each pair of nodes that are connected, and a function can be defined for each numeric function effect and also each action’s duration. The composition for the simple move action in Fig. 19 is shown in Fig. 20.

It is important that the extraction of the actions as a macro do not interfere with the rest of the model, as otherwise the resulting macro could prevent valid plans being discovered. For a more general discussion on the restrictions on composition, refer to [71]. We have identified certain additional effects, and conditions, that our abstraction approach supports. For example, we allow a structure indicating an asset is idle. In this case, a proposition associated with the moving asset is deleted at the start of the move action, and then added again at the end. We also allow static numeric effects, where these are not used in conditions of any actions, which is a common property for functions used for defining metrics.

**Fig. 21:**
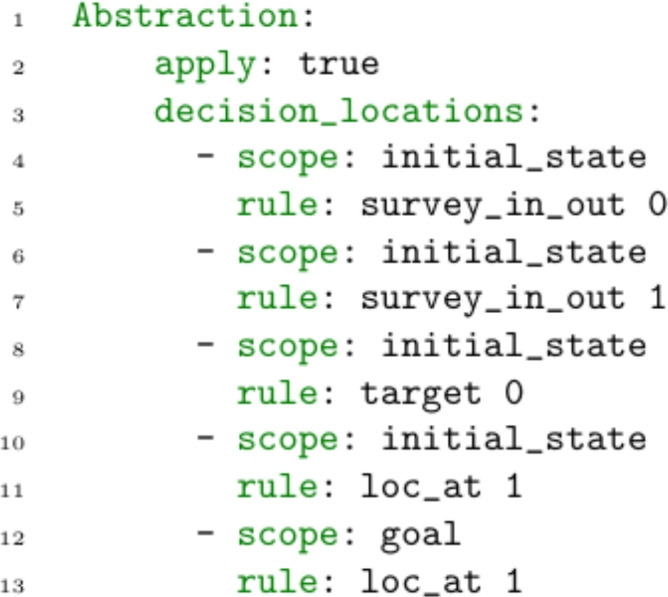
An extension to the domain interpretation in the YAML language. The specification allows for decision locations to be identified using initial state and goal rules.

*Important Points for Navigation* In many navigation tasks the locations can be divided into locations that actions must be taken for the completion of the task (e.g., the initial position of an UAV, part of a survey, or a location to be targeted), and those that are visited only as part of navigation. Our abstraction therefore involves abstracting the transit paths, so that the decision points in the problem are limited to this set of locations where decisions are made (we call these required locations). As a consequence, omitting important locations from this set can lead to pruning (all) solution plans.

In our approach, we modify the definition of the abstraction above by removing nodes from *MapNodes* before creating the shortest paths. The result is that the macro actions can represent sequences of move actions that transition through all of the nodes in *MapNodes*, but that the navigators can only transition between the restricted set of nodes. The important locations are identified from the predicates in the scenario’s initial state and goal. Figure 21 presents the abstraction specification for the UAV domain. In this scenario, the important points are the location of targets, surveys, and the launch and recovery locations. In the specification we specify the initial location of the assets with the fourth rule. The rule indicates that the scope is the initial state, that the predicate is loc_at, and the object is the first parameter.

In order that the user can query actions in the model at the lowest level, the selection of these locations is automatically extended with any locations important to the operator’s queries, as necessary. The appropriate abstraction is therefore created each time the planner is run in the context of the user queries. As a consequence the planner solves a reduced problem, while allowing the user to query specific elements of a plan’s routing.

**Fig. 22:**
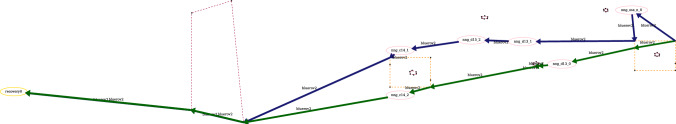
The figure compares the route planning for two metrics. The green line presents the path generated when minimising total-time, and the blue line illustrates the path chosen when minimising the duration near wind turbines. The paths are both optimal with respect to the criteria. The green line is direct. The blue line avoids the wind turbines, including a large deviation at the beginning of the path.

*Recovering the Plan* Given a solution to the abstracted plan, generating the associated plan in the original model requires replacing the composed move actions with the appropriate sequence of individual move actions. This can be done by retrieving the sequence of actions used to build the connection map.

In this work when generating the sequences of original actions, we select the sequence that minimises the impact on the metric function. In Fig. 22 we plot two paths generated for different metrics. Note, that both of these paths are optimal, with respect to each of the particular criteria. The green line illustrates a path generated to minimise total-time. The green line is direct, and passes near to two wind turbines. The blue line illustrates the path chosen when minimising the duration near wind turbines. The blue line avoids the wind turbines, and includes a large deviation at the beginning of the path (see far right).

*Encoding User Constraints* In order to modify the abstraction to support the user’s queries, the set of decision nodes (*MapNodes*) is extended with the locations from the user queries.

## User guided plan exploration

We build on the work in [7] to support user queries for our autonomous vehicle scenario. In  [7]—introduced in the background section—user queries related to ground actions are used to generate contrastive explanations. In their work, they focus on the queries identified in [1] as interesting for planning in general. We consider a similar plan space exploration approach, but we extend the set of query types with specific examples for the autonomous vehicle scenario. In this section we consider queries over the resource allocation, over numeric functions, and new commands for introducing concepts into the planning model. We then illustrate the use of the additional concepts and these new types of interaction in our UAV scenario.

### Numeric fluent queries

In  [7], the focus was on the properties of specific actions in a plan. However, in our context, it is natural to ask queries ranging across other problem structures, including numeric functions. For example, in the context of UAVs, maintaining communication can become important. This is particularly challenging in the context of missions in the proximity of wind turbines, which can lead to interference in communications. As a consequence, we have extended the work in [7] to also support additional model structures, including numerical variables.

In the context of propositional fluents, although there are still some decisions to make, the construction of appropriate constraints is relatively unambiguous. However, in the case of numeric fluents, this problem is less straightforward. Let us consider the query template: *“Why is F so high [at time t]?”*, for numeric fluent *F*, and (optionally) time *t*. The direct application of the approach would be to ensure that the fluent was simply lower (not the same) in the contrastive plan (at the appropriate time). However, this can result in plans that are very similar, and the impact on the numeric fluent can be minimal.

As a consequence, we propose the following query template: *“Why is F not lower by Z% [at time t]?”*, for numeric fluent *F*, percentage *Z*, and (optionally) time *t*. In this case, the user can indicate the appropriate interpretation of the situation. In particular, they can be precise about the percentage decrease that would make a meaningful difference in this situation. For example, *“Why is the depth of BlueROV1 not lower by 10% at time 800?”*

*Incorporating the User Query into the Model* We follow the same steps as in [7] to generate the contrastive explanation. In particular, their approach first builds a hypothetical model (HModel) and uses it to generate a hypothetical plan (HPlan). The HModel is made by adding structure to the existing model. In this case with an intention of constraining the plan space to only accept plans where the value of *F* is lower than the indicated value at the specified time. To achieve this we introduce additional structure, including a time window and a new action to enforce the constraint on *F*.

We start with the current model $P=\langle F,A,I,G,\mathcal {O},T\rangle$, and define the HModel as $HP=\langle F',A',I,G',\mathcal {O},T'\rangle$, where:

- $$F'=F \,\cup \,\{ open\_window , constraint\_satisfied \}$$
  - $open\_window$, indicates when constraint checking is permitted
  - $constraint\_satisfied$, records that a constraint was successfully checked
- $$A'=A \,\cup \,\{ record\_constraint\_satisfied \}$$
  - $record\_constraint\_satisfied$, an action that records that the constraint was satisfied
- $$G'=G \,\cup \,\{ constraint\_satisfied \}$$
- $$T'=T \,\cup \,(\{ open\_constraint\_window ,t), (\lnot open\_constraint\_window ,t+\epsilon )\}$$

And with action $record\_constraint\_satisfied$ (as $a_c$) specified as:

$$\begin{aligned} \textrm{dur}(a_c)&= 0 \\ \textrm{cond}_{\vdash }(a_c)&= \{ open\_constraint\_window ,\ F < v \} \\ \textrm{cond}_{\leftrightarrow }(a_c)&= \emptyset \\ \textrm{cond}_{\dashv }(a_c)&= \emptyset \\ \textrm{eff}_{\vdash }(a_c)&= \emptyset \\ \textrm{eff}_{\dashv }(a_c)&= \{ constraint\_satisfied \} \end{aligned}$$

The value of *v* is calculated by simulating the original plan and observing the value of *F* at time *t*, and reducing its value by *Z*%.

*Explanations for* ***“Why F high?”*** *Queries* Our explanations have several parts. First we use the HModel to generate an HPlan. This HPlan is then compared against the original plan, in order to generate a contrastive explanation. Part of this explanation is in the direct comparison of the planning actions, as was done in [7]. We have also generated visualisations based on this comparison (see Fig. 7 and Sect. 8). There is also a textual explanation that compares the plans in terms of performance or optimisation criteria, which takes the form:

**Table:** 

If we reduce *F* [at time *t*] then:	
$\bullet$ In terms of the optimisation criteria $\mathcal {O}$: **The plan becomes ...**.	

The explanation for the example above is:

**Table:** 

If we reduce the depth of BlueROV1 at time 800 then:	
$\bullet$ In terms of the optimisation criteria total-time: **The plan becomes 591.993 units worse**.	

### Optimisation query

In the existing work on plan space exploration, the focus has been on forcing trajectory constraints into the model to ensure that generated plans exhibit some property. The operator has not been able to query the optimisation criteria. However, it is clear within our scenario that several factors might be interesting to an operator while planning a mission, and that exploring this is just as important as plan features. For example, in our scenario, two factors that were suggested were: communication cost and risk.

To support queries about the optimisation function, our system supports the following query: *“Why do you not minimise F?”*, which allows the operator to query why a specific criterion was not used. In response to this query, the system will of course create a new plan that minimises *F*. However, this query can be seen as an indication that the user considers this an important feature for assessing the plan. As a consequence, the system will also update the list of objective functions (more below).

*Setting Model Objective*

This type of query asks why some property is not true of a plan. Following the approach for standard queries, we first identify a corresponding model modification so that the property must be true in any future plans. In this case, the specific property is objective function is to minimise *F*. As a consequence, the model modification requires that we set the objective function instead to minimise *F*. In fact, we augment this function with a tiebreaker of the existing function.

In the first instance, the optimisation function is to minimise *total-time*. If the user asks to minimise a function *communication-cost*, then the optimisation function becomes: (minimise (+(communication-cost) (* (total-time) (SmallM))).

*Incorporating the User Query into the Model* We again specify the HModel by adding structure to the existing model. In this case the intention is to change the model’s optimisation function.

We start with the current model $P=\langle F,A,I,G,\mathcal {O},T\rangle$, and define the HModel as $HP=\langle F,A,I,G,\mathcal {O}',T\rangle$, where:

- $$\mathcal {O}'=\mathcal {O}_2 + M \cdot \mathcal {O}_1 \quad \text {with} \quad M \ll 1$$
  - This is defined for the new optimisation function $\mathcal {O}_2$, and the original optimisation function $\mathcal {O}=\mathcal {O}_1$
  - And for *M*, a small positive constant ($M \ll 1$) that ensures $\mathcal {O}_2$ is prioritised, and $\mathcal {O}_1$ is used to refine the selection among solutions with equivalent or near-equivalent $\mathcal {O}_2$ values.

*Explanations for Multiple Objective Functions*

As formalised in [7], constraints can be composed, and the user can chain together queries to explore through the plan space. In the initial node of this search, it is assumed, as is the case in our scenario, that the optimisation criteria is to minimise *total-time*. At this node, we initialise a set of objective functions and populate it with the *total-time* criteria. When the user queries the optimisation criteria, we add the criteria *F* to the list of objective functions. In the example above, the list would have *communication-cost* (with the tie-breaker), and *total-time*.

In the original explanations, the objective function was implicit. However, in the context of more than one objective function, we can present the impact of each objective function, allowing a better understanding of the comparison. The explanation takes the form:

**Table:** 

If we minimise this function then:	
$\bullet$ In terms of the optimisation criteria $F_0$: **The plan becomes ...**.	
$\bullet$ In terms of the optimisation criteria $F_1$: **The plan becomes ...**.	
$\bullet$ ...	

The explanation for the example above is:

**Table:** 

If we minimise this function then:	
$\bullet$ In terms of the optimisation criteria total-time: **The plan becomes 801.999 units worse**.	
$\bullet$ In terms of the optimisation criteria communication-cost, with TIE BREAKER: total-time: **The plan becomes 602.726 units better**.	

### Resource management query

We consider a query type that we support in order to allow the user to question a plan’s use of resources. The query allows the user to suggest an alternative preferred allocation: “Why not allocate NE to T*[, and NE to T*]?”, for navigator entities (*NE*), and task lists ($T*$). An example valid query is *“Why not allocate BlueROV1 to Survey0 and Survey1, and BlueROV2 to Survey2?”* As demonstrated in Subsect. 6.2, this query can be implemented directly using the force allocation function.

*Incorporating the User Query into the Model* In this case the intention is to constrain the plan space to only accept plans where a certain set of resource assignment has been used to service some of the tasks. To achieve this we introduce additional structure, to ensure the tasks are achieved using the appropriate assets.

We start with the current model $P=\langle F,A,I,G,\mathcal {O},T\rangle$, and define the HModel as $HP=\langle F',A',I,G',\mathcal {O},T\rangle$, where:

- $$F'=F \,\cup \,\{ progress\_marker_0 ,..., progress\_marker_n \}$$
  - $progress\_marker_i$, indicates that the *i*-th task has been completed appropriately.
- $$A'=A_{T} \,\cup \, A_{\lnot T}$$
  - $A_{T}$, are the task satisfaction actions, and each are extended with the appropriate set of progress maker effects (see below).
  - $A_{\lnot T}$, are any other actions, copied directly from *A*.
- $$G'=G \,\cup \,\{ progress\_marker_0 ,..., progress\_marker_n \}$$

The task satisfying actions are identified as described in in Subsect. 6.2. Each task satisfying action $\textit{TSA}_\textit{i}\in A_T$ is specified for original action *a*, as:

$$\begin{aligned} \textrm{dur}(TSA_i)&= \textrm{dur}(a)\\ \textrm{cond}_{\vdash }(TSA_i)&= \textrm{cond}_{\vdash }(a) \\ \textrm{cond}_{\leftrightarrow }(TSA_i)&= \textrm{cond}_{\leftrightarrow }(a) \\ \textrm{cond}_{\dashv }(TSA_i)&= \textrm{cond}_{\dashv }(a) \\ \textrm{eff}_{\vdash }(TSA_i)&= \textrm{eff}_{\vdash }(a) \\ \textrm{eff}_{\dashv }(TSA_i)&= \textrm{eff}_{\dashv }(a)\, \cup \{ progres\_marker_i \} \end{aligned}$$

*Explanations for* *“Why not this resource allocation?”* *Queries* Our explanations have several parts. First we use the HModel to generate an HPlan. This HPlan is then compared against the original plan, in order to generate a contrastive explanation. Part of this explanation is in the direct comparison of the planning actions, as was done in [7]. We have also generated visualisations based on this comparison (see Fig. 7 and Sect. 8). There is also a textual explanation that compares the plans in terms of performance or optimisation criteria, which takes the form:

**Table:** 

If we force the resource allocations $T*$ then:	
$\bullet$ In terms of the optimisation criteria $\mathcal {O}$: **The plan becomes ...**.	

The explanation for the example above is:

**Table:** 

If we force the resource allocations *BlueROV1 to Survey0 and Survey1*, and *BlueROV2 to Survey2* then:	
$\bullet$ In terms of the optimisation criteria total-time: **The plan becomes 1191.003 units better**.	

Notice that forcing resource allocations can reduce the decision problem, which can help certain times of planning and lead to shorter planning times. In this case, it has also led to the planner finding a shorter plan.

### User-guided language extensions

Extending the model with the concepts in the MAST language could unnecessarily impact the computational cost of the model. We therefore allow the user to add concepts to the model. We use the input template *“Monitor function_description”*, where *function_description* describes one of the functions of the MAST language. As demonstrated in the previous section, each of the concepts supported in the MAST language is associated with a function that can operate the model extension on the underlying planning model.

*The Total Distance Navigated by a Navigator* The first language extension is built over the distance travelled function specified in the previous section. We support user commands of the form: *“Monitor the total distance travelled by NE”*, where *NE* is a navigator entity. The result of this command is that the system will call the distance travelled model extension MAST function. The function will extend the planning model with a new function distance_travelled_by_*NE* in the planning model. The domain model is automatically updated (as described in the previous section) so that the function accumulates during planning.

*Incorporating the User Command into the Model* In this case the intention is to add a new total distance accumulation function into the model. To achieve this we introduce additional structure, to ensure the appropriate accumulation.

We start with the current model $P=\langle F,A,I,G,\mathcal {O},T\rangle$, and define the HModel as $HP=\langle F',A',I,G,\mathcal {O},T\rangle$, where:

- $$F'=F \,\cup \,\{ distance\_travelled\_by\_NE \}$$
  - $distance\_travelled\_by\_NE$, is a numeric fluent that accumulates the distance travelled by *NE*.
- $$A'=A_{DT} \,\cup \, A_{\lnot DT}$$
  - $A_{DT}$, are the distance incurring actions for *NE*, and each are extended with a distance accumulating effect (see below).
  - $A_{\lnot DT}$, are any other actions, copied directly from *A*.

Determining the set $A_{DT}$ is as described in Subsect. 6.1. Each distance accumulating action $\textit{DTA}_\textit{i}\in A_{DT}$ is specified for original action *a*, as:

$$\begin{aligned} \textrm{dur}(DTA_i)&= \textrm{dur}(a)\\ \textrm{cond}_{\vdash }(DTA_i)&= \textrm{cond}_{\vdash }(a) \\ \textrm{cond}_{\leftrightarrow }(DTA_i)&= \textrm{cond}_{\leftrightarrow }(a) \\ \textrm{cond}_{\dashv }(DTA_i)&= \textrm{cond}_{\dashv }(a) \\ \textrm{eff}_{\vdash }(DTA_i)&= \textrm{eff}_{\vdash }(a) \\ \textrm{eff}_{\dashv }(DTA_i)&= \textrm{eff}_{\dashv }(a)\, \cup \{ distance\_travelled\_by\_NE \mathrel {+}= edge\_distance_{XY} \} \end{aligned}$$

Where *X* and *Y* are the start and end locations for action *a*.

As an example, in our system, we can add a numeric variable that accumulates the total distance travelled by BlueROV1, with this command: *“Monitor the total distance travelled by BlueROV1”*. The result is a new function distance_travelled_by_bluerov1, which is incorporated in the planning model, and properly maintained, allowing this value to be used in future queries.

*The Duration that a Navigator is near a Location* Another common concept in UAV problems relates to the distance between objects during the plan. In this work, we focus on the distance between a navigator and a static structure. We support user commands of the form: *“Monitor the duration that NE is near to LE”*, where *NE* is a navigator entity, and *LE* is a location entity. Similar to the above, the result of this command, is that the system will call the *duration near* model extension MAST function. The function will extend the planning model with a new function duration_*NE*_near_*LE*. The domain model is also automatically updated (as described in the previous section) so that the function accumulates during planning.

*Incorporating the User Command into the Model* In this case the intention is to add a new duration near accumulation function into the model. To achieve this we introduce additional structure, to ensure the appropriate accumulation.

We start with the current model $P=\langle F,A,I,G,\mathcal {O},T\rangle$, and define the HModel as $HP=\langle F',A',I,G,\mathcal {O},T\rangle$, where:

- $$F'=F \,\cup \,\{ duration\_NE\_near\_LE \}$$
  - $duration\_NE\_near\_LE$, is a numeric fluent that accumulates the duration that *NE* is near *LE*.
- $$A'=A_{DN} \,\cup \, A_{\lnot DN}$$
  - $A_{DN}$, are the actions where *NE* is near to *LE* for some proportion of the duration, and each are extended with a duration accumulating effect (see below).
  - $A_{\lnot DN}$, are any other actions, copied directly from *A*.

Determining the set $A_{DN}$ is as described in Subsect. 6.1. Each duration accumulating action $\textit{DNA}\_\textit{i}\in A_{DN}$ is specified for original action *a*, as:

$$\begin{aligned} \textrm{dur}(TNA_i)&= \textrm{dur}(a)\\ \textrm{cond}_{\vdash }(TNA_i)&= \textrm{cond}_{\vdash }(a) \\ \textrm{cond}_{\leftrightarrow }(TNA_i)&= \textrm{cond}_{\leftrightarrow }(a) \\ \textrm{cond}_{\dashv }(TNA_i)&= \textrm{cond}_{\dashv }(a) \\ \textrm{eff}_{\vdash }(TNA_i)&= \textrm{eff}_{\vdash }(a) \\ \textrm{eff}_{\dashv }(TNA_i)&= \textrm{eff}_{\dashv }(a)\, \cup \{ duration\_NE\_near\_LE \mathrel {+}= \\&\quad \quad \quad \quad \quad \quad \quad \quad \quad proportion\_edge\_near\_LE_{XY} * \textrm{dur}(a)\} \end{aligned}$$

Where *X* and *Y* are the start and end locations for action *a*.

As an example, in our system, we can add a numeric variable that accumulates the duration that BlueROV1 is near to target1, with this command: *“Monitor the duration that BlueROV1 is near to target1”*. The result is a new function duration_bluerov1_near_target1, which is incorporated in the planning model, and properly maintained, allowing this value to be used in future queries.

*Labelled Object Collections*

In Sect. 5, we described how MAST structures supported labelling collections of navigating and location entities, such as the collection of points that form an exclusion zone, or all of the points that represent wind turbines. The commands above can use any navigator or location entities in their arguments. These commands therefore provide a flexible mechanism for generating language extensions in the planning model. For example, we can add a numeric fluent to monitor the duration that all the vehicles are near to any of the points of exclusion_zone_1 with the command *“Monitor the duration that* **the vehicles** *are near to* **exclusion_zone_1** *”*.

### Properties of user queries

The queries introduced here share several properties with those presented in [7]. In particular, under a bounded planning time, a response can always be generated, even if that response merely reports that no solution was found within the allotted time. In the case of overconstrained problems, it would also be possible to use an existing approach that explains why a problem is unsolvable [72], or using an approach targeting overconstrained problems [10].

As in [7], user queries may lead to solution pruning and can even render a problem unsolvable. For instance, a query may require that an asset travel a shorter distance than is physically necessary to move from its initial to its goal position. In the case of queries that change optimisation criteria, there is no added constraint and therefore the plan space is not changed.

The constraints and model extensions are also composable. For example, it is possible to add a function to monitor the distance travelled by BlueROV1, add a constraint to it, and subsequently manage the resource allocation of both BlueROV1 and BlueROV2. In our formulation, all newly introduced constraint names are parameterised by query depth, ensuring that the symbols introduced at each layer remain disjoint.

Finally, the user commands defined above (i.e., those for adding functions) are limited in scope: they can introduce new functions and add positive effects over these functions, so they cannot alter the existing plan space. In their case, as with the optimisation queries, there might be changes in performance due to simulating the additional model structure.

### Use of the queries for UAV scenarios

We consider the example additional operator objectives presented in Subsect. 3.3 to demonstrate how the approach presented in the previous sections allows the construction of appropriate queries. In the next section we will explain how user interaction is managed in our task-bot interface.

*Objective 1:* To examine the impact on the plan if we minimise the distance travelled by BlueROV1, we can first create a numeric fluent to accumulate the distance travelled by that vehicle. We can then set the optimisation function to minimise this function. This can be done in our system using these commands: *“Monitor the total distance travelled by BlueROV1.”* and *“Why do you not minimise the total distance travelled by BlueROV1?”*

*Objective 2:* The second objective considered resource allocation. The operator can use a resource allocation query to reallocate the objectives. For example, using the query: *“Why not allocate BlueROV1 to Survey0, and Survey1 and BlueROV2 to Survey2?”*. As a result the system extends the model with features to monitor the completion of tasks, and the required goals to ensure the correct assets are used to service each task. This provides very specific control over resource allocation. However, it would be possible to add a function that counts the number of tasks serviced by each asset. This could be achieved automatically using similar machinery, and would allow queries to target the number of allocations, which might allow the user to better express their queries.

*Objective 3:* The third objective considered risk. The system supports allowing the operator to introduce functions to monitor the proximity between dynamic and static objects. This would allow us to consider queries regarding risk around certain areas of the scenario, such as, whether BlueROV1 is near to ExclusionZone1. For example, we can ask in the system: *“Why do you not reduce the duration that BlueROV1 is near to ExclusionZone1?”*

*Objective 4:* To examine the impact on the plan if we avoid the wind turbines, we can first create a numeric fluent to accumulate the duration that the vehicles are near the wind turbines. We can then set the optimisation function to minimise this function. This can be done in our system using these commands: *“Monitor the duration the vehicles are near to the wind turbines.”* and *“Why do you not minimise the duration that the vehicles are near to the wind turbines?”*

## A task-bot for explainable planning

Our explainable planning module can be used as a standalone system, and operates from a MAST specification. It also forms part of an end-to-end mission planning and execution system, which has been used to control robot missions in a real quarry (the scenario is presented in Fig. 1). In this section we overview the main system modules, and describe the task-bot interface, which allows users to interact with the explainable planning component.

**Fig. 23:**
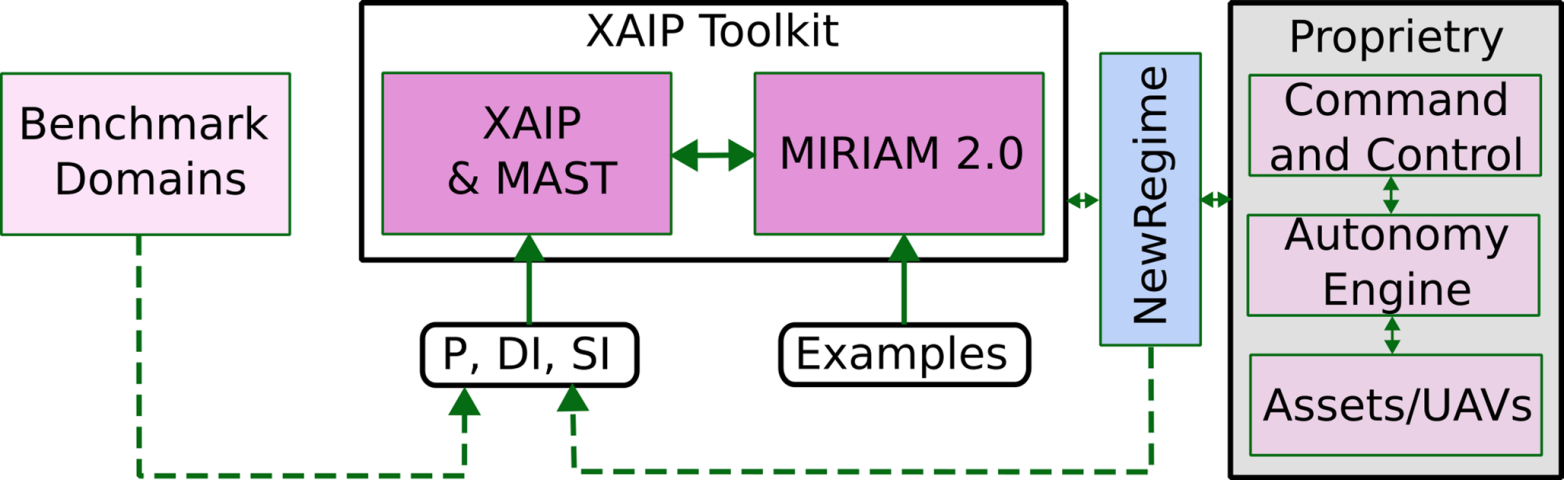
System architecture diagram. The XAIP toolkit operates from a MAST specification: $\langle P, DomainInterpretation, ScenarioData\rangle$, and sets of example queries.

### System architecture

Our system architecture is presented in Fig. 23. The XAIP toolkit (middle) operates from a MAST specification: $\langle P, DomainInterpretation, ScenarioData\rangle$, and sets of example queries. There are two modes for using the system: either as a standalone system, or within a end-to-end mission planning and execution system. In each case the toolkit relies on the appropriate specification files and a PDDL model. The specifications for two benchmark domains is described in Sect. 9. Within the planning and execution system, our implementation builds on proprietary software 69, 73 and their components: command and control, which is the operator interface for planning and monitoring and the autonomy engine, which is embedded onboard the assets that can run the mission. Three new components: *NewRegime*, *MIRIAM 2.0*, and XAIP & MAST (see Fig. 23), have been specifically designed to provide support and effective assistance to a human *operator* in planning and observing UAV mission scenarios.

The system architecture consists of several parts. At the bottom layer are the assets themselves, i.e., the UAVs with their specific hardware and vehicle control autonomy software. *Neptune* 69 is a mission level autonomy engine, which includes software embedded onboard the asset that can run the mission by providing low-level actions, tasks, and behaviours to the vehicle control and is able to adapt the initial plan dynamically to the environment. *SeeTrack* 73 is command and control software, providing a map based user interface supporting the *operator* during mission planning, allowing the *operator* to specify launch, recovery, survey, and target objectives, and other important structure for the mission, including exclusion zones. It also generates an initial mission plan for Neptune, which is built with feedback loops for the operator to visualize how the assets would behave to meet their objectives. Using this feedback, operators can then adjust the mission plan until, eventually, the mission plan is uploaded to the software on the assets. The interface can also be used to monitor a mission in progress based on feedback provided by Neptune.

NewRegime acts as a middleware component between the existing system and the new components. It provides (gRPC) interfaces to apply programmatically, similar functions *operators* would apply through the *Topside* user interface (e.g., adding or removing objectives, or allocating resources). Additionally, NewRegime allows extra or external information relevant to the mission specification (e.g., locations of hazardous areas such as sandbanks) to be injected 74 . This allows for (parts of) the mission specification process to be automated or supported by other systems or information sources.

On top of NewRegime, we developed an entirely new task-bot assistant modelled on the MIRIAM (Multimodal Intelligent inteRactIon for Autonomous systeMs) system [75]. MIRIAM 2.0 guides operators in actively shaping the underlying task using planning-oriented interaction (see below).

Finally, the explainable planning component (XAIP & MAST) was developed in order to decouple the new features away from the proprietary software, allowing us to exploit a concise representation of the planning problem, and cleanly separate it from the solver. The main mission elements are extracted through NewRegime from the mission specification (e.g., assets, exclusion zones, objectives, asset launch and recovery points, and transponder positions), and used to define a problem model and MAST scenario specification. The XAIP & MAST component is used to support query interpretation from MIRIAM 2.0, to construct answers to the operator queries, and to generate explanations of the mission plans. Underpinning our approach is the *Optic* planner [76], which is sensitive to alternative metric functions.

### MIRIAM 2.0

MIRIAM 2.0 is a task-bot assistant powered by a production-ready framework known as RASA open source.^2^ While underpinning on Rasa components, MIRIAM takes advantage of Rasa NLU and Rasa Core components. Rasa NLU is the core element for understanding user input. Rasa Core acts as an intelligent decision-making component to estimate the following action based on the interpreted input from the user, the conversation history, and the conversation flow, which can be expressed in the form of rules or learned through stories. MIRIAM seamlessly integrates the XAIP and MAST wrapper components into each task-bot model, starting with intent recognition and ending with selecting the appropriate action in the designed conversation flow and stories. This allows MIRIAM to automatically manipulate the planning model and generate responses as appropriate.

#### Intention recognition

MIRIAM NLU pipeline is based on the DIET (Dual Intent and Entity Transformer), a multi-task architecture for intent classification and entity recognition. The architecture is based on a transformer shared for both tasks. A sequence of entity labels is predicted through a Conditional Random Field (CRF) tagging layer on top of the transformer output sequence corresponding to the input sequence of tokens. For the intent labels, the transformer output for the complete utterance and intent labels are embedded into a single semantic vector space [77].

Four distinct intents were trained to map the user’s intent to the XAIP modules: one intent for all “why” questions, one for adding feature descriptors, one for executing optimisation criteria, and an additional intent that collects various XAIP features enabling actions such as maintaining constraints, restarting the system, displaying the plan, and comparing plans through interaction with the task-bot.

Once the task-bot recognises one of those intents, the system executes the MIRIAM policy for dialogue management. It is based on the Transformer Embedding Dialogue (TED) Policy, a multi-task architecture for next-action prediction [77]. The policy predicts which action to take from the conversation flow and the stories at each iteration. Subsequently, The task-bot action is mapped to an XAIP function or triggers several XAIP behaviours. In addition, MIRIAM collects the complete user message to pass on to the relevant XAIP component as part of executing an action.

Regarding robustness and error handling, in addition to the system’s own intention recognition, MIRIAM 2.0 also employs a strategy to manage fallbacks. This is a two-stage approach, called Two-Stage-Fallback. Suppose the intent estimated by DIET has a probability below a certain threshold. In that case, DIET classifies the intent as $nlu_{fallback}$ and executes an action that asks the user for clarification about the estimated intent, such as, *“Did you mean X?”* If the user answers affirmatively, the TED policy model for that intent is activated. If the response is negative, MIRIAM attempts to clarify by considering the next highest estimated intent. If fallback persists, it simply responds, *“Sorry, I cannot assist you with that query”*.

In the specific case of the related XAIP component, fallbacks sometimes occur not in the DIET or TED estimation, but during the interpretation of the XAIP query. In such cases, MIRIAM provides a breakdown of the query interpretation and displays it on screen for user analysis (e.g., as a framing for the generated explanation text). Then, the operator can continue the interaction, ignore the fallback message, and reformulate the query. Moreover, the user is free to navigate through the constraint tree, managing how and when they are composed (see below). Both DIET and the XAIP query interpreter support multiple variations, enhancing natural interaction. Additionally, after each query, the XAIP toolkit is used to generate example queries, tailored to the context. MIRIAM presents these to the user (including some suggested queries as quick replies), in order to provide assistance of the available queries.

#### Visualization

**Fig. 24:**
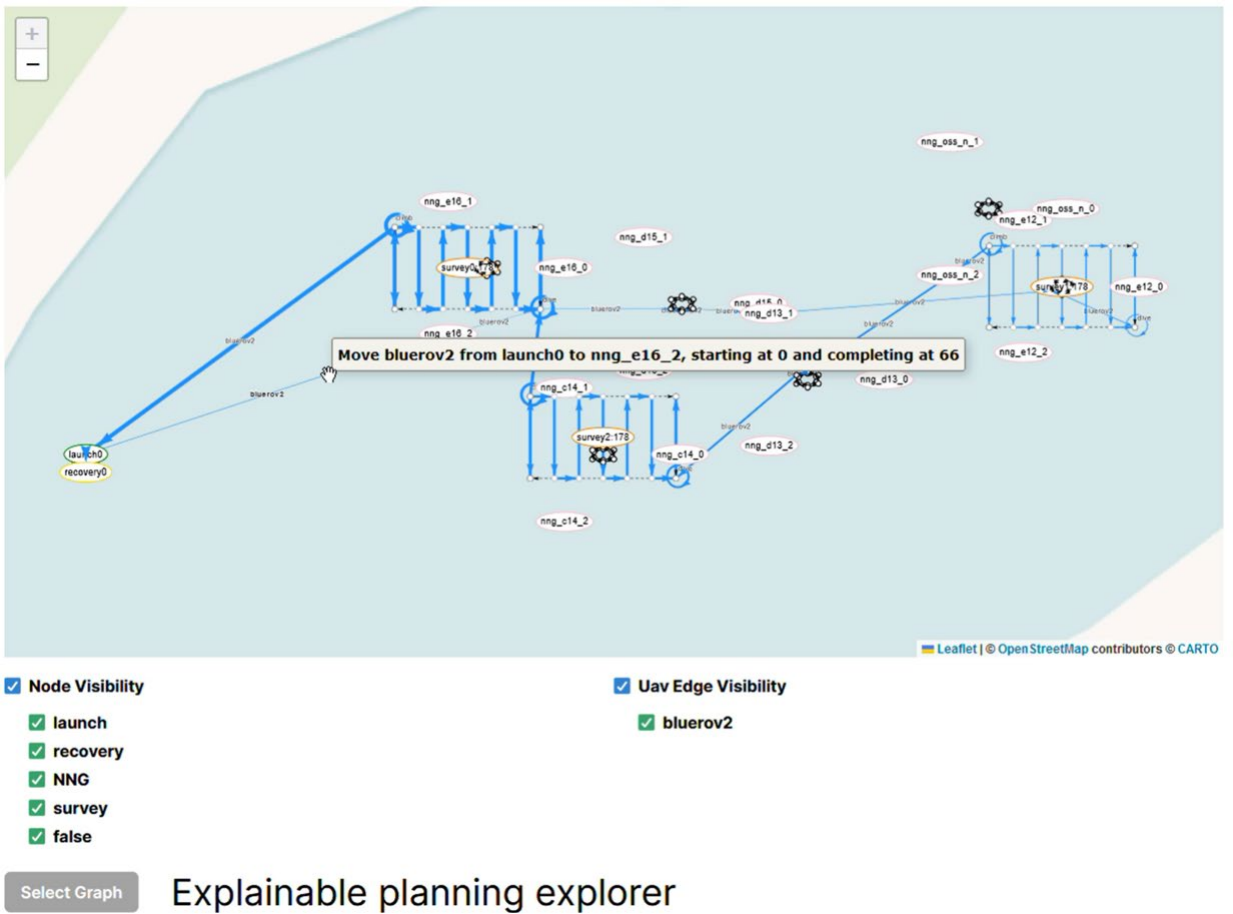
Plan rendering web-based component.

In addition, a web-based interface was designed for user task-bot interaction. A Node.js component was developed to render generated plans as graphs (e.g., see Fig. 24). This component enables the storage and visualization of graphs for each user query and the creation of comparison graphs for different plans. This component takes the visual specification presented in Subsect. 5.2 and builds a visualisation from the structure and plan rules in the domain specification. It provides a straightforward approach for generating graph based visualisations, meaning it works particularly well in navigation type tasks.

The component constructs an interactive visualisation. Users can interact with the edges and nodes of the graph, allowing them to hide or show aspects of the plans. For example, they can visualise the plan for a specific UAV, or switch between viewing the original and the new plans, which facilitate the analysis of the tasks. Additionally, users can hover over each edge to view a description of the action, and double-clicking copies the action definition. This set of mouse interactions is useful for transferring action descriptions to queries.

#### MIRIAM storyline

**Fig. 25:**
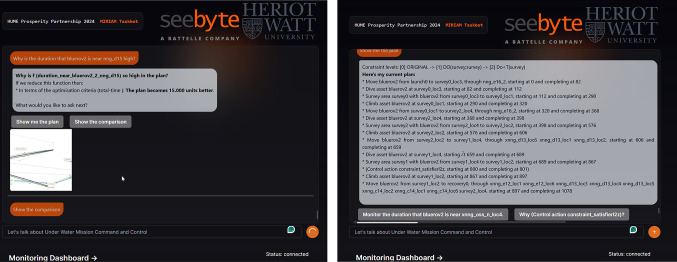
MIRIAM 2.0 web-interface: The web interface shows the different elements it combines to enhance the user experience, such as quick-reply buttons, graph component thumbnail, a constraint level guide, text-based explanations, and a scrollable message history

The interaction between the operator and the task-bot to explore the model space, generate explanations, and develop plans begins when the operator asks MIRIAM to start explaining the mission. At this point, the main components of the mission are extracted, the MAST specification scenario is created, and a plan is generated. Once this information is processed, MIRIAM presents the operator with the initial plan and provides a set of sample queries, generated by the system based on the current specifications (see Fig.25). The users can display the graph of the initial plan or use any of the contrastive queries provided by the system. When the user enters a query, such as *“Why not...”*, the system generates a new plan, and uses the plan and the previous plan in order to generate an explanation. MIRIAM presents the textual explanation, and can then be used to examine the plans separately, or view the comparison visualised. The task-bot MIRIAM always provides textual explanations and access to plans in graphical form after a constraint is explored. However, the user can also request the plans in text form too (e.g., *“Show me the current plan”*).

During the interaction the user controls the building of the composition of the constraints. They can choose to retain the new constraint by asking MIRIAM with an instruction like *“Can you keep this constraint?”*. The operator can also remove the last constraint using a phrase like *“Could you remove the last constraint?”*, or return to the original plan by typing *“Can you return to the original plan?”*. MIRIAM then updates the current model, plan, and the graphical component accordingly.

### Planning structure templates

We have used structure templates to support both mapping between planning structures and usable text, and also to provide candidate sets of example sentences for describing planning structures.

**Fig. 26:**
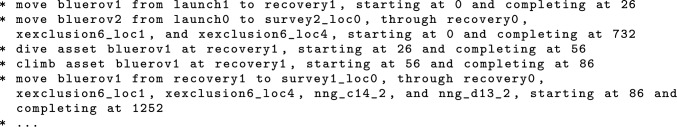
Part of the plan description for the plan visualised in Fig. 6, including collated move action descriptions (action descriptions 2 and 5).

#### Textual representation

Our approach to rendering plans and function descriptions in text follows [23]. It relies associating actions and functions in the planning model with tags detailing verbs/noun and prepositions that might be used in describing the action/function. For example, for the move action, we include ‘move’, ‘navigate’, ‘go’, and ‘drive’ as alternative verbs, and prepositions such as ‘from [the] *from*’, ‘starting at [the] *from*’, and ‘from [the] *?from*’. Following [23], we also support summarising rules, which allow certain chains of actions to be condensed where an effect summarisation exists (e.g., as in the case of sequences of transit actions). Figure 26 presents a plan fragment demonstrating a basic plan description.

#### Structure text reference examples

Our approach for creating examples of textual references for the actions and functions in the model relies on the same annotation approach (see above). The tagging described can be used to generate relevant sets of candidate rules for matching action and function descriptions. As with [23] the verbs are extended using mlconjug3 [78], which can be used to provide alternative verb conjugations, supporting user input in various tenses. These examples are then used in the intention recognition and parameter extraction parts of the system in order to allow effective mapping between user input and planning structures.

## Evaluation

In this section, we first present an empirical evaluation were we examine the effectiveness of the proposed constraints, we compare the use of reduce and minimisation queries, and consider the impact of the map abstraction. We then present a qualitative user study that examines the impact of the ideas presented in the paper on the participants’ use of the system. Finally we discuss the approach and the results, and consider future directions.

### Empirical evaluation

In this part we examine the use of the presented approach in an empirical evaluation, which examines how the numeric queries scale with query and instance complexity, whether the constraints have their intended effect, whether the constraints work in other MAST domains, and how the abstraction impacts on the approach.

To conduct these tests we used the XAIP and MAST component (see Sect. 8), which extends an implementation of [7] with the ideas presented in this paper. The standard approach in the tests is to use the planner to generate an initial plan. Then use the toolkit in order to make some modification to the model, and then compare the plans. In each run (unless stated) we set the planner timeout to 3 min, with a maximum of 8Gb memory.

***UAV Scenarios***

For these tests we have generated three uav problem instances from three case studies.

**Demo**: The first scenario involved two UAVs, three targets, a survey and an exclusion zone;
**Quarry**: The second scenario (quarry) set the scenario in a real quarry, where we tested a single UAV completing the mission. The scenario involved six wind turbines and an additional exclusion zone, and three survey areas.
**Windfarm**: The third scenario uses data from a real wind farm in Scotland, UK. In this scenario we used two UAVs. The scenario involved six wind turbines and an additional exclusion zone, and three survey areas.

We have prepared MAST specifications for each of the three scenarios.

***Benchmark Instances***

 We have also used two benchmark domains with the MAST structure. In particular, we have identified two temporal benchmark domains with navigation behaviours: driverlog and rovers. In driverlog problems, packages are collected and delivered by trucks, which must be driven by drivers. There are two maps: the road network for driving, and the path network for the drivers to walk. We have used the Time problems from IPC3 [79]. In these problems the various drive and walk actions are each associated with a time. In rovers problems, rovers navigate between waypoints, and have certain tasks, such as taking samples, photos, and communicating data through a lander. We have selected the SimpleTime problems from IPC3 [79].

The locations in these problems are not associated with coordinates. In order to create appropriate MAST specifications for these for the problems of these domains we used the following process. We first extracted a connected graph from the problem instance. In the case of driverlog, we use the time to traverse as an indication of the length of the edge. In the case of rovers no length indication was provided. We then used a graph layout engine, which lays out the points using the connections between nodes (and the indicated lengths if appropriate) to influence their positions. This provides a specific coordinate for each location. These are used to generate the scenario data specification for the problem. The MAST specifications for an instance of the rovers and driverlog benchmark domains are presented in Appendix A.

We use the benchmark set for the first part of the study and then we have selected three problems of increasing size from each domain to test our approach. For driverlog we have selected pfiles 3, 10, and 14, and 3, 10, and 18 for rovers.

**Fig. 27:**
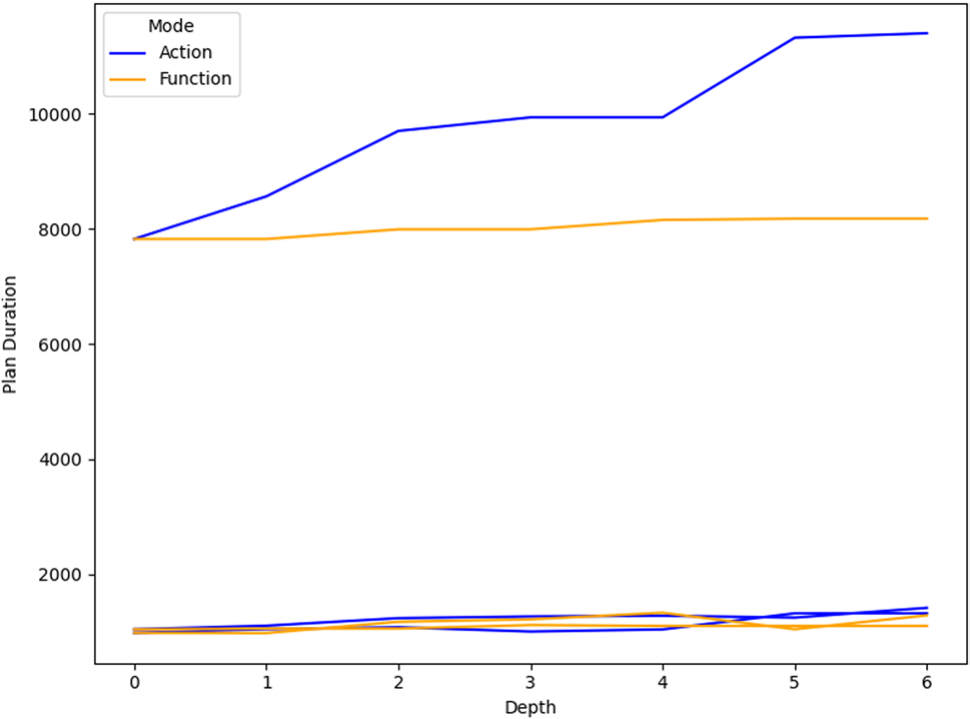
For instances of the uav domain and query types (Action or Function), plots plan duration against query complexity. The blue lines are used to plot results for Action queries, and the orange lines plot results for Function queries.

**Fig. 28:**
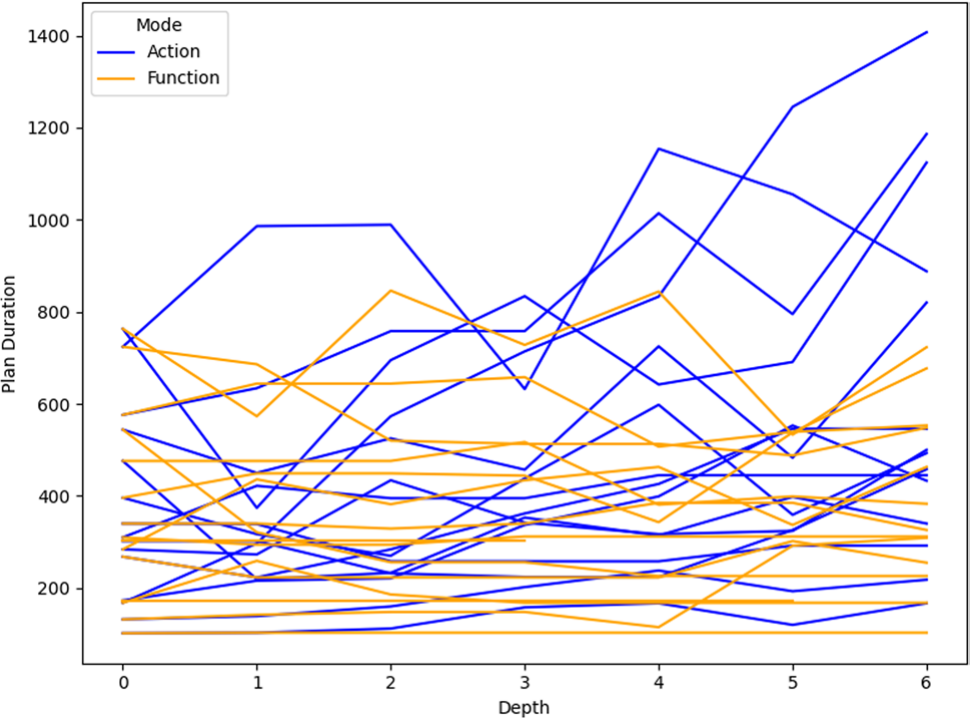
As above for instances of the driverlog domain.

**Fig. 29:**
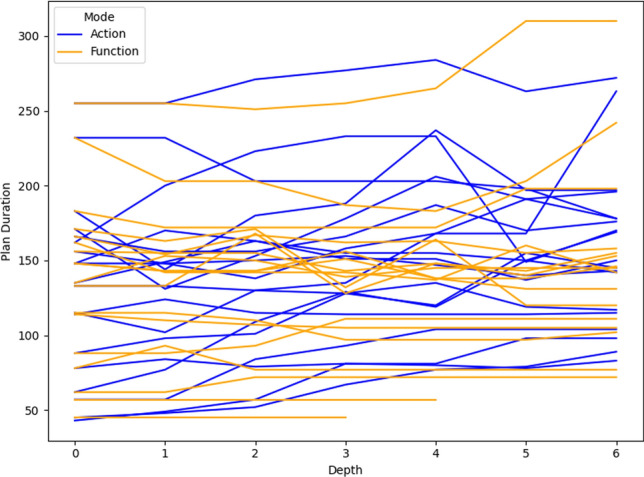
As above for instances of the rovers domain.

**Fig. 30:**
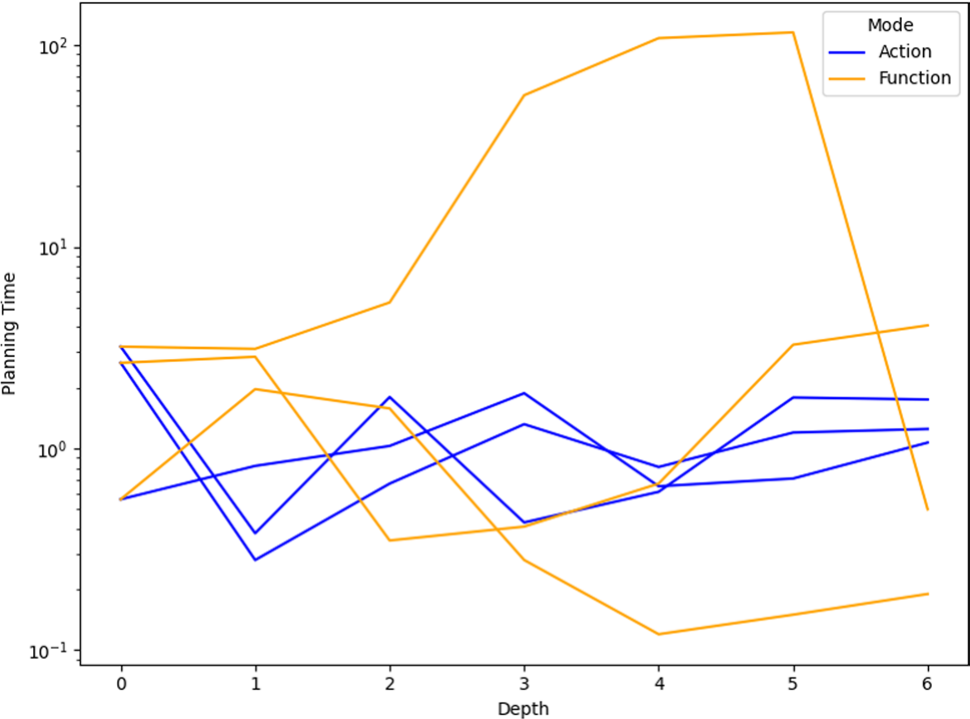
For instances of the uav domain and query types (Action or Function), plots planning time against query complexity. The blue lines are used to plot results for Action queries, and the orange lines plot results for Function queries.

**Fig. 31:**
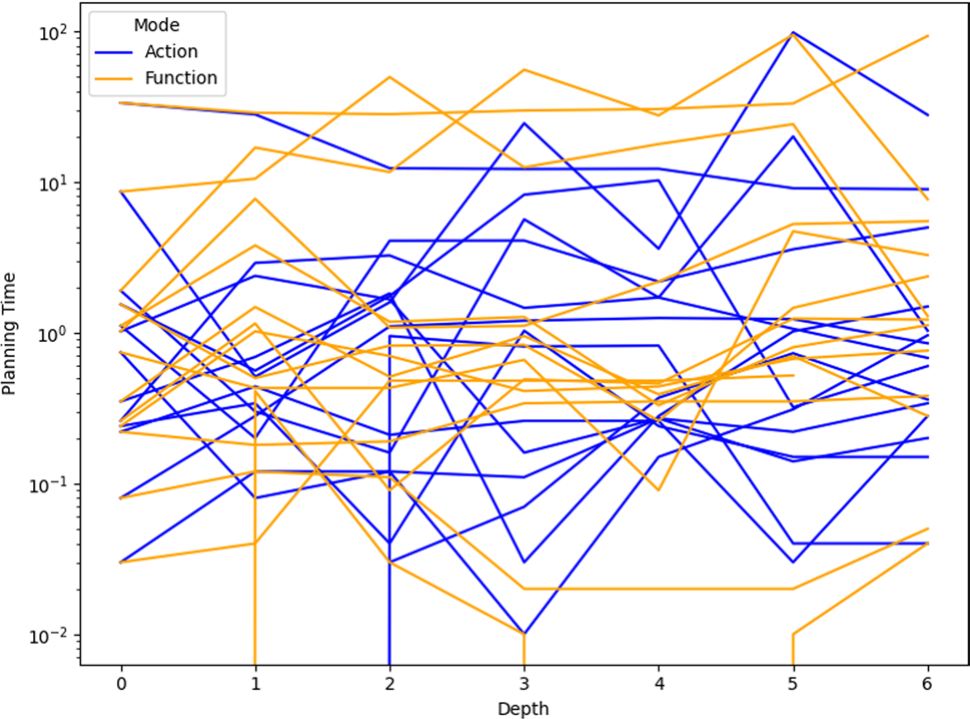
As above for instances of the driverlog domain.

**Fig. 32:**
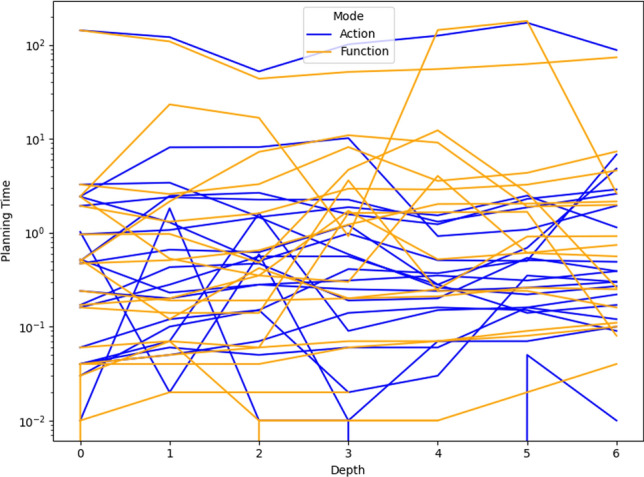
As above for instances of the rovers domain.

### Impact of query and instance complexity

We first examine the impact of query and instance complexity on performance. To do this we use each of the benchmark instances for which *Optic* returns an original plan in the available resources (3 min and 8Gb memory). We aim to compare the impact of the numeric constraint (*‘Why is F so high?’*) denoted *Function*, against the original action based queries (*‘Why A?’*, *‘Why not A?’* and *‘Why A before B?’*) denoted *Action*. As we are interested in considering plan duration, as well as planning time, we do not consider queries that change the optimisation criteria. We consider this in later sections. In the context of numeric functions, the constraint will typically involve both a MAST concept extension (e.g., *“Monitor the total distance...”*), and a numeric function. These two steps are counted as a single step when we plot depth.

For each instance and set of query types (functions or actions) we aim to build a constraint of depth 6, by chaining together individual constraints. At each depth we recorded the planning time, and the plan duration. We first consider the impact of query complexity, and then the impact of problem instance size. In some cases, chaining together constraints will lead to failures, and we therefore also examine these failure cases.

#### Impact of query complexity

Figures 27,  28, and  29, plot lines for each instance and query type. The lines plot the total plan duration for increasing constraint complexity (depth 0 to 6). Notice, the depth 0 indicates the plan duration of the original plan. The blue lines are used to plot results for Action queries, and the orange lines plot results for Function queries.

The results demonstrate that there is a general upwards trend, which is expected. As additional constraints are added to the model, plans are pruned, and in some cases these will be the shorter plans. Of course, due to the satisficing planning approach adopted, in some cases the planner will happen upon a shorter plan. The action queries appear to have more impact on the plan duration than the numeric functions. In particular, the impact of numeric functions tends to lead to the plan duration remaining constant, or gradually increasing with constraint depth. This can be expected, as whereas the action constraints prune certain actions, the numeric constraints instead limit the accumulation of certain values. The results demonstrate that the plan duration is more changeable as the instance size increases. This is likely due to the greedy planning approach adopted. In particular, the Action constraints lead to high plan durations in larger driverlog instances.

Now considering planning time, Figs. 30,  31, and  32, plot lines for each instance and query type. The lines plot the total planning time (plotted on a log scale) for increasing constraint complexity (depth 0 to 6). Notice, the depth 0 indicates the planning time for the original plans. The blue lines are used to plot results for Action queries, and the orange lines plot results for Function queries. There is less pattern in the planning times. Although the times tend to vary around similar times. In particular, there are only some instances where there are consistent behaviour as the constraint depth increases. It appears that some of the constraints assist with search discovering a plan and others do not.

**Table 3:** Failure type and counts across domains and XAIP or MAST query types. Reports: number of instances with no queries, and average number of deadends and failures (per attempted query)

Domain	Query	# No queries	Avg. deadends	Avg. failures
UAV	Action	0	0.0556	0.167
UAV	Function	0	0.0	0.389
Rovers	Action	0	0.333	0.088
Rovers	Function	3	0.0	0.088
Driverlog	Action	0	0.222	0.067
Driverlog	Function	2	0.0	0.022

*Analysis of Failures* During the random composition of the constraints, there were several failure cases. In these cases, we recorded the failure and attempted to continue. There are three types of failure that were encountered:

No Queries The approach used to generate the next query could not find a valid query to apply;
Deadends An added query led to the problem becoming impossible to solve;
Failures The planner failed to find a solution to the problem within the available resources.

Table 3 presents the failure results during the generation of the constraints for the above results. The table records the number of situations where no queries could be discovered, the average number of deadends (averaged over the number of query generation steps), and the average number of failures.

In each of the domains, the results show that there are more likely to be deadends in the original query types. This is perhaps to be expected, as the function queries can often be satisfied by reducing the accumulation of a function, whereas the action queries prevent/constrain those actions. In particular, in some of the smaller instances, several of actions will be necessary, and have no possible alternatives.

We have drawn a clear distinction between the deadend and failure cases. In reality, this is a heuristic estimate. In particular, planners like *Optic* are effective at detecting deadends. However, this is not complete. As a consequence, some of the reported failures might be deadends. However, as the planner cannot detect the deadend and attempts to find a solution in search, their impact on interaction follows the table as reported. In the benchmark domains there are relatively few failures. In the uav scenario there are relatively more failures. This is perhaps due to the relative power of the constraints. In the uav domain we have defined additional location collections (e.g., individual and collective wind turbines), which will have wider implications on the plan. In fact, several of these cases in uav involved several duration near constraints, which in combination might have been difficult, or impossible to solve.

For three of the rovers and two driverlog problems, no viable function queries were found. This can occur in small instances with simple topology, where the rover never moves from its starting location. For this study we assume that duration near is accumulated during movement, which is typically a reasonable heuristic approach. See Subsect. 6.1 for how we can also register duration near for idling, and other non-movement actions. If the vehicles never moves, all of these distance, and duration near functions are zero, and as such, it is impossible to find applicable MAST queries.

**Fig. 33:**
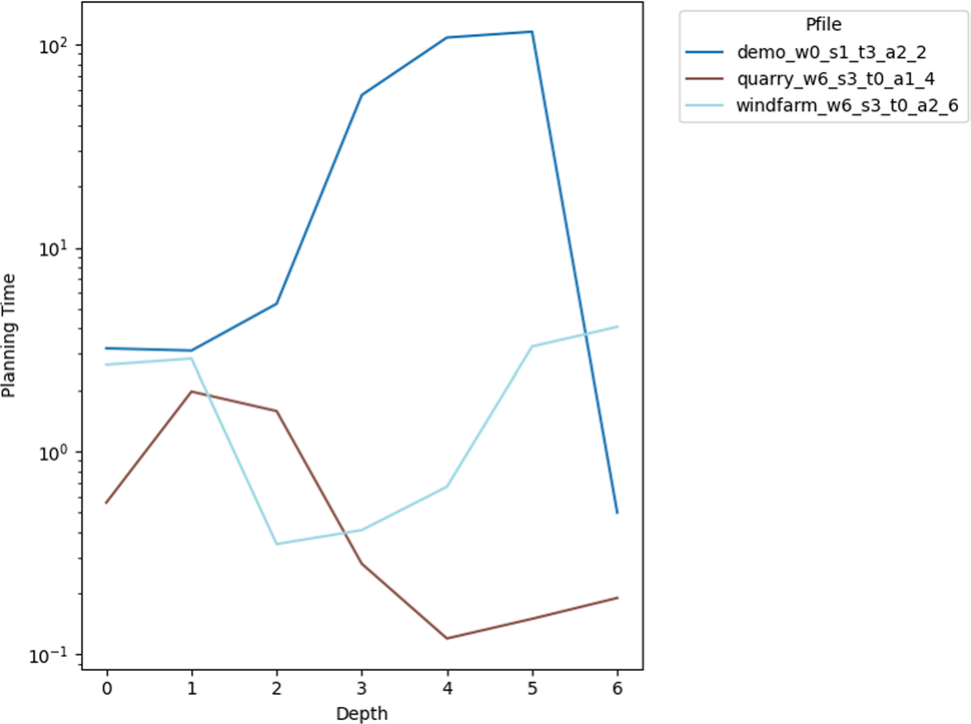
For instances of the uav domain and Function query types, plots planning time against query complexity. The lines are coloured to distinguish problem instances.

**Fig. 34:**
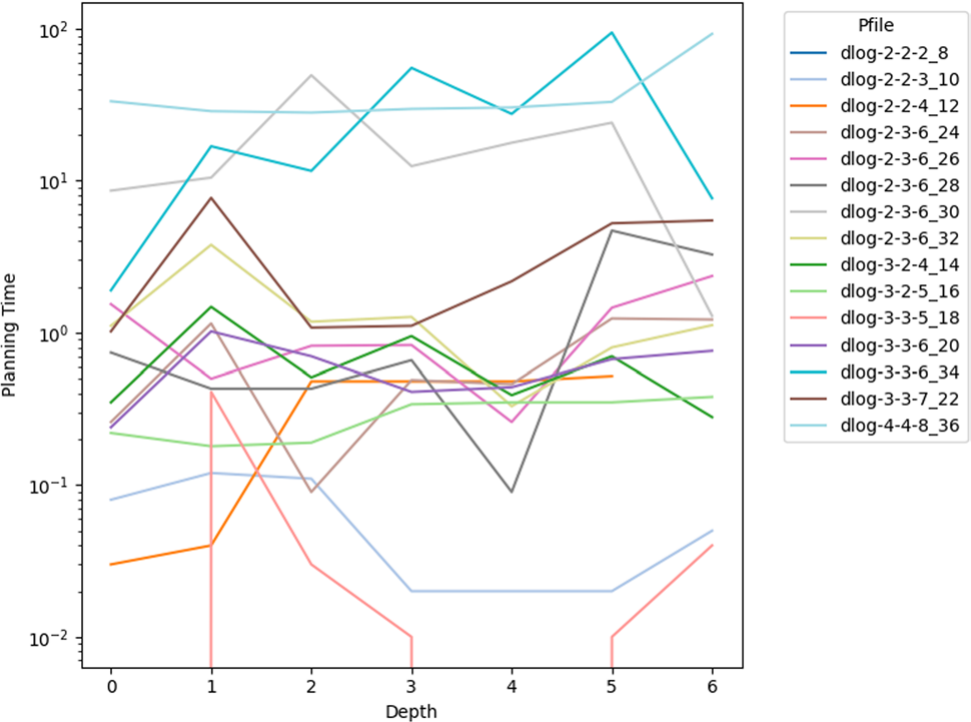
As above for instances of the driverlog domain.

**Fig. 35:**
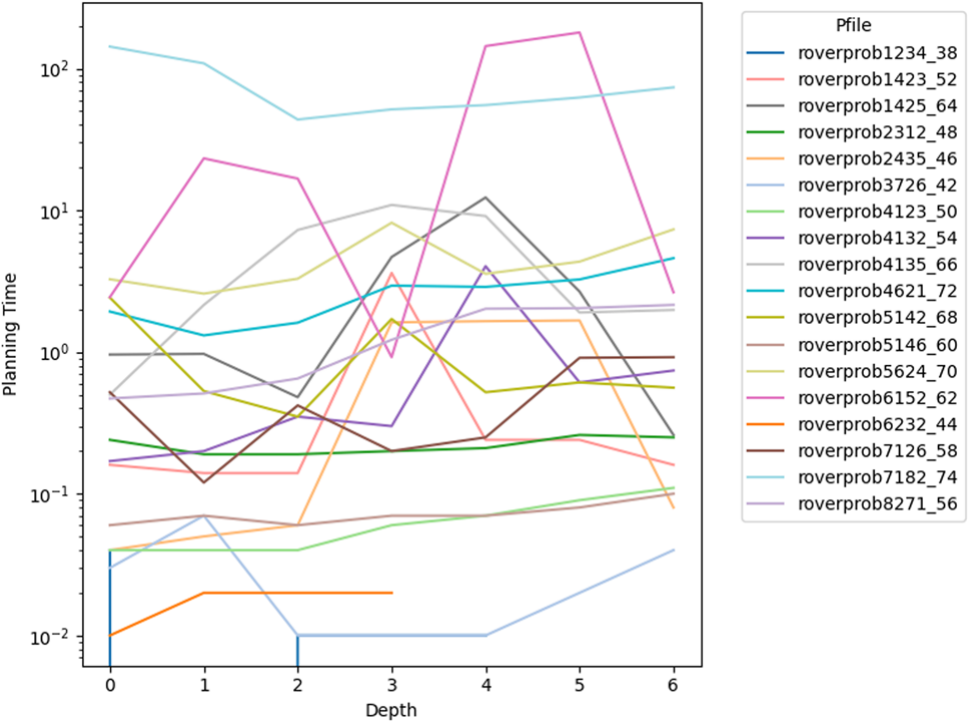
As above for instances of the rovers domain.

**Table 4:** Table reporting number of actions (|*A*|) and objects (|*O*|), and number of planning actions in the original plan, for problem instances used in study

Domain	Problem	#Actions	#Objects	#Action in $\pi$
uav	demo	322	21	37
uav	quarry	1260	72	24
uav	windfarm	2852	93	45
rover	roverprob1234	63	13	10
rover	roverprob4213	53	14	10
rover	roverprob3726	76	16	12
rover	roverprob6232	86	18	8
rover	roverprob2435	144	18	25
rover	roverprob2312	178	19	44
rover	roverprob4123	151	20	23
rover	roverprob1423	328	25	35
rover	roverprob4132	362	27	42
rover	roverprob8271	382	29	44
rover	roverprob7126	436	27	37
rover	roverprob5146	366	28	25
rover	roverprob6152	749	30	62
rover	roverprob1425	525	31	43
rover	roverprob4135	751	32	66
rover	roverprob5142	671	33	56
rover	roverprob5624	1227	44	69
rover	roverprob4621	1837	50	59
rover	roverprob7182	3976	60	150
driverlog	dlog-2-2-2	88	11	8
driverlog	dlog-2-2-3	108	14	21
driverlog	dlog-2-2-4	120	14	12
driverlog	dlog-3-2-4	144	16	24
driverlog	dlog-3-2-5	168	16	21
driverlog	dlog-3-3-5	222	16	13
driverlog	dlog-3-3-6	252	18	15
driverlog	dlog-3-3-7	270	19	40
driverlog	dlog-2-3-6	384	22	48
driverlog	dlog-2-3-6	516	26	30
driverlog	dlog-2-3-6	616	29	27
driverlog	dlog-2-3-6	948	39	62
driverlog	dlog-2-3-6	1148	48	42
driverlog	dlog-3-3-6	1158	37	49
driverlog	dlog-4-4-8	2592	51	105

#### Impact of instance size

Figures 33,  34, and  35, replot lines for each instance and Function query types. The lines plot the total planning time for increasing constraint complexity (depth 0 to 6). This time, the lines are coloured by instance, to allow comparison of the impact on easier/harder instances. Table 4 presents the number of planning actions (|*A*|), the number of objects (|*O*|), and the number of planning actions in the original plan for each instance.

In general, for the benchmark domains the smaller instances take less planning time to find a plan, and the larger instances take longer to plan. Although not consistent, the plot reveals a general slightly upwards trend in planning time with constraint depth. As well as increased time due to the constraints, part of this increase is likely associated with the increase in model complexity. In particular, these steps are typically associated with a model extension, which may lead to an increase in time to simulate the model.

Interestingly, the highest time to plan line in uav is actually in the smallest scenario. This perhaps demonstrates clearly the importance of *Optic*’s initial search phase. Once it starts its best-first search, it is unlikely to return a plan within the requirements for interaction. In this case, the problem is small enough that even when using best-first search it manages to discover an appropriate solution. It is possible that a similar situation in one of the larger strategies would have led to a failure (within the resource limits).

We noted for the plan duration results, that the larger instances were associated with more changeable plan durations. Notice that this variation will impact on the quality of the generated explanations. Notice that this is due to properties of the planning approach used in the study and not due to the approach. However, given the complexity of the planning problem, and particularly the interactive nature of the XAIP-as-a-service approach, there exists an important trade-off between efficient planning and plan quality, especially as the instance complexity increases.

### Impact of language extensions

We now investigate how well the language extensions operate in uav scenarios, and then across three MAST domains.

#### Effectiveness of constraints in UAV scenarios

We first examine the proposed language extensions, and their use with queries, and investigate their performance in the uav scenarios.

**Table 5:**
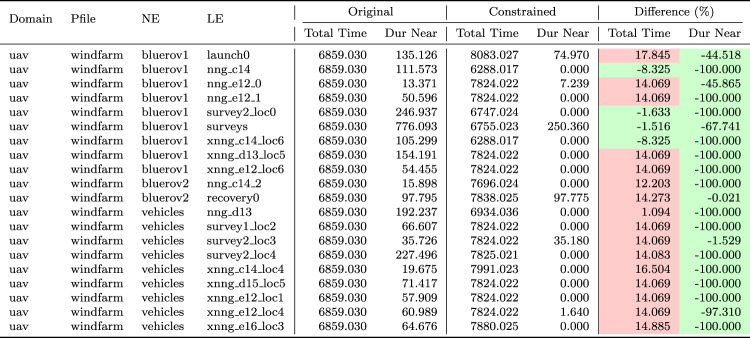
The table plots total time for the original model, the constrained model, and the percentage difference; and the same for the duration that *NE* is near to *LE*.

The percentage difference is coloured to reflect if the constrained model improved (green), made worse (red), or did not change (grey) each metric

*Monitoring Duration Near* In this part we look at the duration near queries. We start by solving the original problem. (Original) We then add a duration near monitoring function, and use it within a minimisation term (Constrained), in order to examine its effectiveness.

For the windfarm scenario we have examined randomly sampled 20 navigation entity (*NE*) and location entity (*LE*) pairs. For each of the 20, the model is extended with a duration near monitor function for the specific navigation and location entities. On requesting this function, the system automatically extends the model with the necessary structure required to monitor the appropriate duration near as it accumulates. The process is explained in detail in Subsect. 6.1, where we presented the structure of the move action after the query *“Monitor the duration that the vehicles are near to the windturbines”* (Fig. 13), the supporting data in the problem model (Fig. 14), visualisations of the model’s view of the proportion near data (in Fig. 12), and a trace of the total distance over time when simulating a plan (Fig. 15).

In order to examine the use of these functions in practice, we have used the monitoring functions within optimisation queries (e.g., *“Why not minimise the duration that vehicles are near to windturbines?”*). In this case, we heavily bias the optimisation term to the new function (0.999), and use the time taken as a tie-breaker (0.001).

Table 5 plots the comparison between plans generated for the original and intervention model, where the intervention minimises the duration near for a specific *NE* and *LE* pair. The table compares the total time and the duration near for the original and constrained models. The difference is also presented in order to emphasise the trade-off between the metrics. In most of the cases the plans for the constrained case are worse than the original plans in terms of time. This is perhaps expected, as we are introducing a new optimisation criteria, and only using time as a tie-breaking term. In contrast, in all cases, the planner finds plans that improve the duration near metric for the respective navigation and location entities. In many cases (14/20), the planner found a plan where the vehicles were never near to the wind turbines (during moving actions).

**Table 6:**
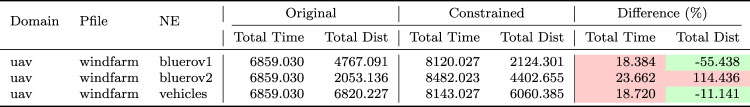
The table plots total time for the original model, the constrained model, and the percentage difference; and the same for the total distance travelled by the NE.

The percentage difference is coloured to reflect if the constrained model improved (green), made worse (red), or did not change (grey) each metric

*Monitoring Distance Travelled* In this part we look at the distance travelled queries. Again, we start by solving the problem without the added structure (Original). We then consider the use of a monitoring function with an minimisation term (Constrained), in order to examine its effectiveness.

For the windfarm scenario we have examined each of the possible navigation entities (*NE*s). For each *NE* we extend the model with a distance travelled monitor function. On requesting this function, the system automatically extends the model with the necessary structure required to monitor the appropriate distances travelled as they accumulate. The process is explained in detail in Subsect. 6.1, where we presented the structure of the move action after the query *“Monitor the total distance travelled by BlueROV1”* (Fig. 9), the supporting data in the problem model (Fig. 10), and a trace of the total distance over time when simulating a plan (Fig. 11).

In order to examine the use of these functions in practice, we have used these monitoring functions within optimisation queries (e.g., *“Why not optimise the distance travelled by BlueROV1?”*). In this case, we heavily bias the optimisation term to the new function (0.999), and use the time taken as a tie-breaker (0.001).

Table 6 plots the comparison between plans generated for the original and intervention model, where the intervention minimises the distance travelled by a specific *NE*. The table compares the total time and the total distance for the original and constrained models. The difference is also presented in order to emphasise the trade-off between the metrics. In all cases the plans for the constrained case are worse than the original plans in terms of time. This is perhaps expected, as we are introducing a new optimisation criteria, and only using time as a tie-breaking term. In the case of BlueROV1 and the vehicles, the planner finds plans that improve the total distance travelled for the respective navigation entity. In the case of BlueROV2, the plan found (within the time limit) is also worse in terms of this metric. We will examine this further below.

**Table 7:**
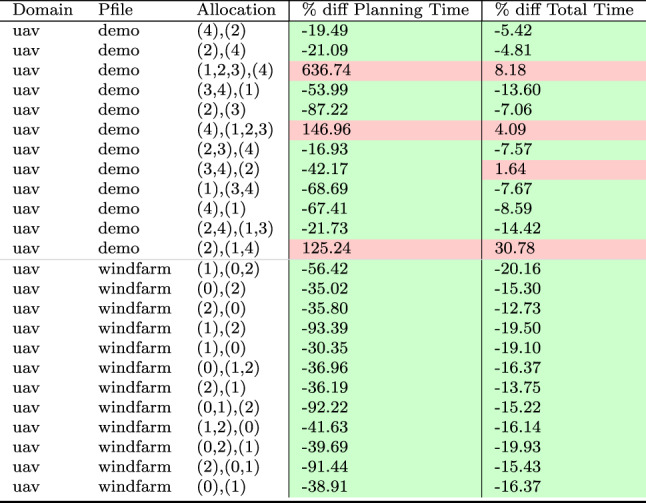
The table plots the percentage difference for planning time (first plan), and total time–comparing the original plan with the plan generated after a resource allocation query.

*Resource Allocation Queries* In this part we look at the resource allocation queries. We started by generating plans for the unmodified model (Original). We then asked queries based on random allocations (Constrained), in order to examine its behaviour in practice.

In order to examine the use of these queries, we have generated random allocations and generated new plans in the demo and wind farm scenarios (these are the scenarios with more than one asset). To do this we gathered the set of tasks and assets and generated the candidate set of allocations. We then sampled 12 allocations in each scenario. For each allocation, the model was constrained using a resource allocation constraint. The system automatically extended the model with the necessary structure required to monitor the satisfied allocations, and added appropriate goals to match the allocations. The process is explained in detail in Subsect. 6.2, where we describe the process, demonstrating its impact from the original plan (Fig. 1) to the plan generated for the constrained model (Fig. 2). We have used the planner’s first plan (rather than a time limit) to generate plans in this context. This allowed us to observe whether adding these constraints have impact on the time taken to find a plan.

We present the results in Fig. 7. The table shows the allocation, and percentage differences in planning time and plan duration. The allocation in the table presents a tuple for each asset, which indicates each asset’s task allocation. The table shows that in this domain, that providing even part of the allocation can assist the planner, which finds a plan quicker in all but three instances. In these scenarios it finds better plans in many cases too (20 out 24). This would not be expected in general, and is more likely to do with the original plans being of relatively poor quality.

*Application to MAST Domains* In this section we demonstrate that the approach that we have developed in this work is applicable to MAST domains. In order to get a broader impression of the use of the queries we have aggregated different queries in each scenario. In each case, a function was created and minimised, as above. We then calculated the difference between the original and new plans. These were then aggregated for each scenario.

**Table 8:**
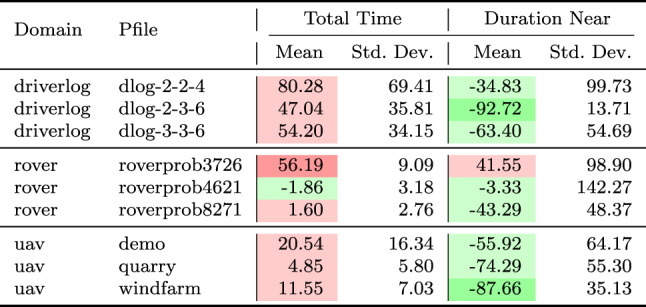
The table presents aggregated duration near results for uav, driverlog and rovers problems.

For each problem, 20 (or all) navigation and location entity pairs were randomly selected. We used the NE and LE to generate a new duration near function, and created a new plan by minimising this function. The table presents the mean and standard deviation of the percentage difference between the original and constrained models, in terms of the two metrics (total time, and the associated duration near function). The mean difference is coloured to reflect if the constrained model improved (green), made worse (red), or did not change (grey) each metric

**Monitoring Duration Near** First we present aggregated data for duration near functions. For each problem, we have randomly selected 20 (or all) pairs of navigation and location entities. Each of these entity pairs was then used (as above) to generate a new duration near function, which was then minimised. We then calculated the difference between the original and new plans. These were then aggregated for each problem. In Table 8 we have presented aggregated duration near results in the three MAST domains. As can be expected, the results show that on average the minimisation of duration near typically leads to worse total time. However, the results show that in most problems, the optimisation of duration near functions leads to improvement in the relevant duration near function. In the case of distance travelled, we are heavily relying on the planner to find a plan that better optimises our metric function. In contrast, much of the improvement in terms of reducing duration near is determined by the selection of transit paths. This is largely abstracted away from the planner, and uses optimal paths (with respect to the optimisation criteria). Consider for example Fig. 22, which illustrates two alternative paths generated for moving the asset to its recovery point using different metrics. It demonstrates that in refining an abstract plan, paths that remove most of the duration near can be found (in this case by avoiding wind turbines). Notice that in rover problems (especially the smallest), the maps are smaller than in the other domains. Moreover, most of the points are determined as important points for navigation (see Subsect. 6.3). As such this abstraction is less effective in rovers. We explore the impact of the abstraction below.

**Table 9:**
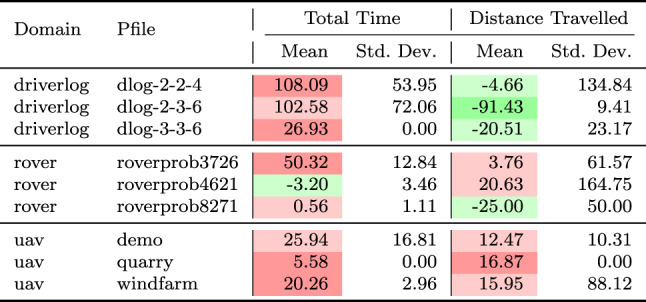
The table presents aggregated distance travelled results for uav, driverlog and rovers problems.

For each problem, 10 (or all) navigation entities were randomly selected. We used the NE to generate a new distance travelled function, and created a new plan by minimising this function. The table presents the mean and standard deviation of the percentage difference between the original and constrained models, in terms of the two metrics (total time, and the associated distance travelled function). The mean difference is coloured to reflect if the constrained model improved (green), made worse (red), or did not change (grey) each metric

**Monitoring Distance Travelled**

For each problem, we have randomly selected 10 (or all) of the navigational entities. Each of these entities was then used as above to generate a new distance travelled function, which was then minimised. We then calculated the difference between the original and new plans. These were then aggregated for each problem. In Table 9 we have presented aggregated distance travelled results in the three MAST domains. As we saw above, the results show that on average the minimisation of total distance typically leads to worse total time. In driverlog the total distance travelled is improved on average. However, in the rovers and uav domains, optimising total distance typically leads to worse performance in terms of distance travelled. In our tests, the returned plans are often some of the first discovered, and potentiall not properly optimised. It is possible with more resources the planner would come up with a better plan. It is also possible that in some of these cases that reducing the total distance is difficult. For example, in the case of trying to optimise the distance travelled by all of the rovers. It is clear that the rovers must still move to service their various tasks. In these cases the altered metric valuation is leading to poorer performance. We have investigated different balances between the new and old optimisation criteria, but with limited impact. We investigate the alternative numeric query types below.

**Table 10:**
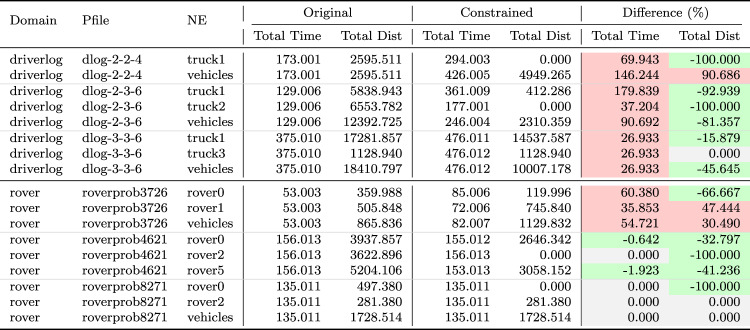
The table presents total distance query comparison results for driverlog and rovers problems.

It plots total time for the original model, the constrained model, and the percentage difference; and the same for the total distance travelled by the NE. The difference is coloured to reflect if the constrained model improved (green), made worse (red), or did not change (grey) each metric

To further examine these results we present results for rovers and driverlog for individual instances. For each instance we randomly generated 3 (or all) *NE*s. These *NE*s were then used to generated a new distance travelled function, Table 10 presents results for the two domains. It plots the comparison between plans generated for the original and intervention model, where the intervention minimises the distance travelled by a specific *NE*. The table compares the total time and the total distance for the original and constrained models. In all driverlog problems the plans for the constrained case are worse than the original plans in terms of time. As before, this can be expected, as we are introducing a new optimisation criteria. As suggested by the aggregated data, the planner is often able to find plans that improve the total distance travelled for the respective navigation entities in driverlog problems. In rovers the performance appears worse in the smaller problems where there are few rovers, and small maps. The new plans found in the larger problems appear mostly similar to the original plans. It appears that in some situations, the new optimisation function is not able to influence the planner to find better plans within the time limit.

**Fig. 36:**
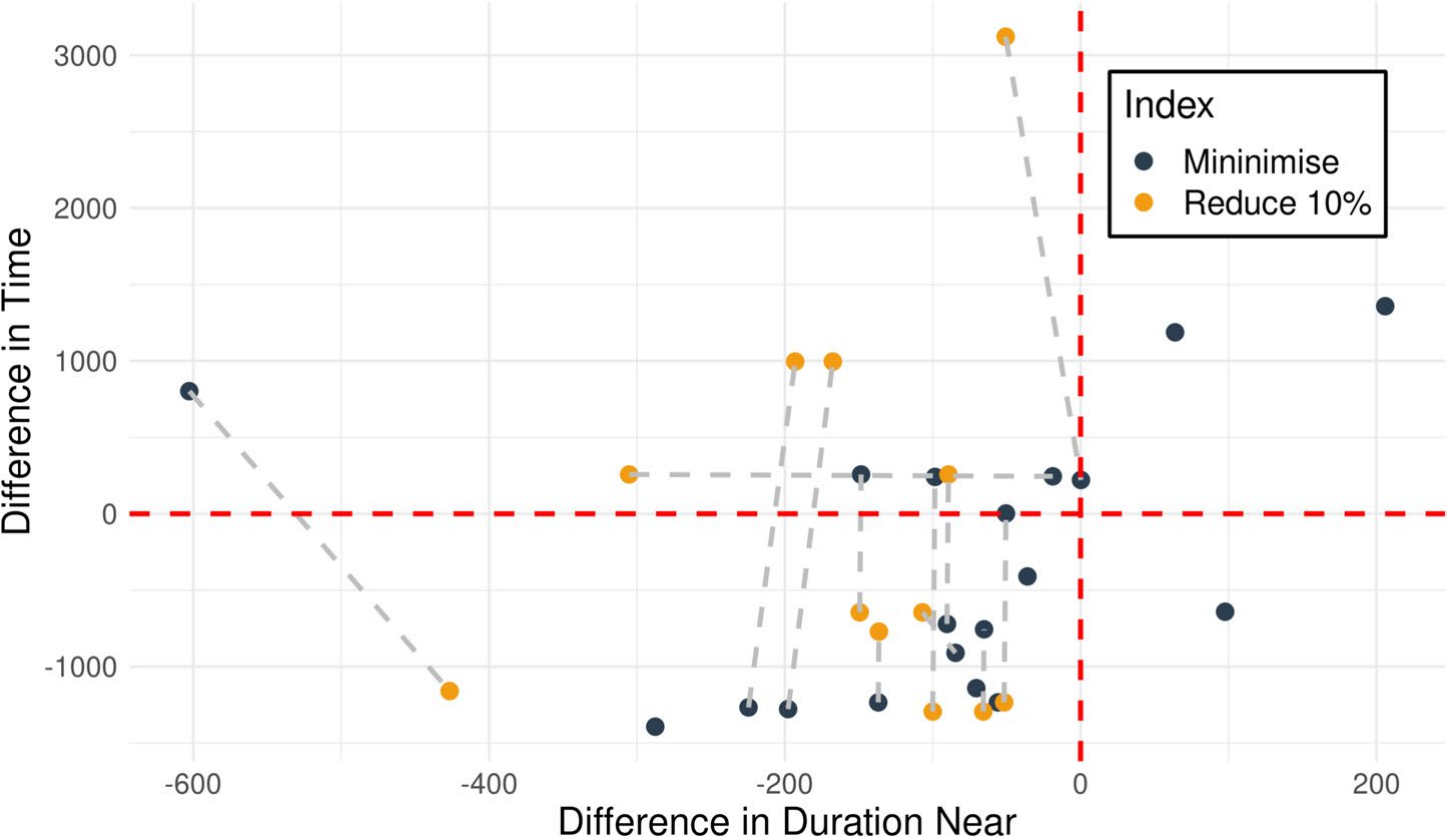
A comparison of using numeric fluent (Reduce 10%) and optimisation (Minimise) queries on the new language structure. Random duration near functions were generated (see text) and the queries were used with the generated function. The difference is plotted between the newly generated plan and the original plan for the two query types and for two metrics (time and the new function).

### A comparison of numeric query types

In this part, we investigate the use of reduce *F* (see Subsect. 7.1) and minimisation (see Subsect. 7.2) query types in the context of added problem structure. We focus on monitoring duration near functions in the windfarm scenario. As before, we randomly selected navigation and location entity pairs (*NE*, *LE*), and used the concept wrapper to generate a new function to monitor the duration that *NE* is near to *LE*. We simulated the original plan, and if it accumulated no duration (in the new function), we disregarded it and selected a new pair. Otherwise, we asked two queries: *“Why not lower F by 10%?”* (Reduce) and *“Why not minimise F?”* (Minimise). We ran the planner to a 60-second timeout for each planning episode. We used a shorter timeout to examine the query performance in a near interactive setting. As a result, we had three plans for each function (subject to the timeout). We then recorded the metric values for both time (the original metric) and the new function (duration near).

Figure 36 compares 20 random functions. Each point is plotted for two metric functions: both total time and the generated duration near function for the run. A dotted grey line joins the two points resulting from the same function. For each query type we plotted the difference observed in these functions between the original and the new plan. As a result, the plot indicates how the two approaches compare in terms of the two functions. Points below the origin indicate an improvement in total time, and points to the left indicate an improvement in the new function. For all but one of the new functions, at least one of the queries generated a plan.

The first observation is that for Minimise, 19 plans were generated, while for Reduce, only 12 of the 20 problems were solved. This can be expected, because the problem might be made unsolvable with the added constraint of Reduce. Also, the added constraint may lead to more failures in *Optic’s* initial search phase, leading to more timeouts (especially with the relatively short timeout). Of course, the use of Minimise does not necessarily mean that the new function will be improved, although it typically does in this scenario. More surprisingly, the two approaches are comparative in terms of their impact on total-time. In Reduce, time is still used to optimise the plan generation, whereas it is only used as a tiebreaker in the Minimise case. The Minimise points that increase the duration near are all associated with failure from the Reduce queries. This suggests that the original plan might have been quite well optimised in terms of duration near. Therefore, although it is likely there will be fewer plans generated, there might be some benefit to combining the approaches. In particular, the plans generated for Minimise that actually have worse metric values (in terms of the criteria indicated by the user), are unlikely to be deemed useful.

**Table 11:**
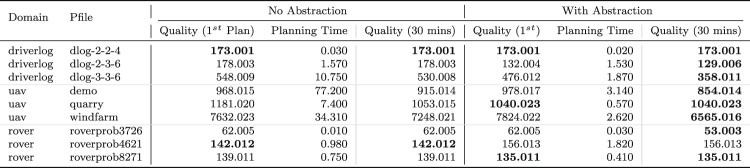
The table presents results for uav, driverlog and rovers problems.

It presents quality (total time) and planning time for solving with no abstraction and with the abstraction

### Abstraction

In Subsect. 6.3 we introduced an abstraction, which has been used in all of the results so far. The abstraction involves abstracting the navigating actions in each domain: the move action in uav and rover, and the drive-truck and walk actions in driverlog. In each domain, the set of important locations (for decision making) is identified. In the case of rover and uav, these are the positions that a vehicle might have to do something (e.g., analyse soil, take a photo). In driverlog, these are initial and goal locations of packages, drivers, and trucks. We now examine the impact of the abstraction on solving time and quality. We do this using the original problems and we test the abstraction in two modes: for first solution (Quality ($1^{st}$ Plan)) and after thirty minutes or the planner hits an 8Gb memory limit (Quality (30 mins)).

Table 11 presents the results. In the no abstraction case, the time taken is variable (0.01 to 77.2 s), and the planner typically takes longer to find a plan. With the abstraction, the planning returns a plan for driverlog and rover problems within 2 s, and within 3.5 s for all problems. In terms of quality of first plans, the results are mixed, with both better and worse quality for with and without abstraction. This demonstrates that the abstraction can be effective. Especially in terms of reducing the first plan time, which is a common use case for our system.

After the time or memory has been exceeded the results for the runs with abstraction are better in eight out of nine cases. It is usually expected that abstraction will reduce quality. In the rover and uav domains, the abstraction does not prevent the planner from finding optimal plans. In driverlog the abstraction can remove optimal plans, although given the domain conventions it will never prune all plans. However, in all of the driverlog and uav problems the quality results are improved with the abstraction. It can be observed that the abstraction is particularly useful in uav and driverlog, which are problems with bigger maps, and fewer important points. These are the problems where the abstraction can simplify the problem. The impact on quality might also be caused by the fact that as part of refining the abstract plan we use optimal paths, whereas the planner can select suboptimal paths between nodes.

### Study design and procedure

The study was conducted as an exploratory qualitative evaluation, composed of participant interaction sessions followed by semi-structured interviews. We used the system as described in Sect. 8, using a model of a real wind farm. Within this setting, participants were tasked with completing three tasks using two versions of the system. For each task, the participant first addressed a problem using only queries available in [7] (Version A) and was subsequently informed of the option to utilize the extended system capabilities supported by the MAST structure (Version B).

Upon completing each task, participants were required to respond to a series of questions to compare the usability of the two system versions. The interviews were recorded and transcribed to gather in-depth insights into participants’ reactions and perceptions of the system; the transcriptions were open coded using an inductive approach [80]; and themes were developed from the codes [81], ensuring a thorough understanding of their experiences.

The three tasks that were used in the study are summarised here:

**Task 1**: The scenario included two UAVs, and three survey area objectives. The initial plan length was 47 actions. Participants were informed that BlueROV2 was an older vehicle with a limited battery capacity, and as a consequence, they were instructed to reduce the vehicle’s travel distance while ensuring the mission’s completion. The initial plan is shown in Fig. 6.
**Task 2**: The scenario included one UAV, and two survey area objectives. The initial plan length was 17 actions. This task focused on BlueROV1. They were provided with a report indicating ongoing maintenance at a certain location, and were instructed to avoid interfering with maintenance operations during the mission.
**Task 3**: The scenario included two UAVs, and three survey area objectives. The initial plan length was 47 actions. Participants were tasked with maintaining good UAV communication. They were instructed that communication performance can degrade when the UAVs operate near wind turbines.

The system was setup as described in Sect. 8, supporting interactions as outlined in section MIRIAM Storyline. The task-bot interface is presented in Fig. 25 and visualisations were presented using the interface as presented in Fig. 24.

In Version A of the system, the system could support queries based on the actions in the model. It could support the following query types:

- Q1: *‘Why is A in the plan?’*
- Q2: *‘Why is A not in the plan?’*
- Q3: *‘Why is A used before B?’*

Where *A* and *B* are action descriptions. For example, a valid query in Version A is *“Why not move BlueROV1 from NNG_D13_1 to Survey0_loc2?”*

In Version B, the system also supported the MAST concepts, and the system could support queries/commands of the following types:

- Q1: *‘Why is A in the plan?’*
- Q2: *‘Why is A not in the plan?’*
- Q3: *‘Why is A used before B?’*
- Q4: *‘Monitor F’*
- Q5: *‘Why not minimise F?’*

Where *A* and *B* are as above, and *F* is a function description. For example, a valid query in Version B of the system is *“Why not minimise the total distance travelled by BlueROV1?”*

*Participants* The study included twelve participants $(N = 12)$, and after no new views were expressed in the final three interviews, saturation was reached and no further participants were recruited. The average age was 29 and the group comprised three female, one non-binary, and eight male individuals. Eight participants had master’s degrees, and four had bachelor’s degrees. Two were master’s students, and ten were pursuing doctoral degrees. Regarding automated planning experience, one reported no experience, four reported they had read or briefly used, seven reported studied or used. All of the participants have either carried out a course including the use of planners, or are doing a PhD. in a related field (e.g., robotics).

### Results

In this part we use the themes to present the results of the study. *Benefit of Contrastive Explanations* Participants found the contrastive mechanism useful when solving Task 1 (version A). The queries that version A provides were sufficient not only to compare plans but also to manipulate them. For example, one participant said, *“You are explicitly questioning why it is taking something you do not want it to do, and then it is giving you an alternative. So it is useful.”* Another participant said, *“It would be easier for many people than trying to go into the plan itself and edit it”*, and similarly, *“It will help people like that a lot rather than trying to figure out what is causing the problems manually.”* This supports the findings of existing work, e.g., [6–8], which has found that users find contrastive explanations useful.

*Power to shape objectives* In Task 1 (version B), participants found that the version B queries allowed them to represent constraints directly associated with the proposed task. In particular, the minimisation query was useful in Task 1. One participant stated, *“You’re more defining the objectives than the plan.”* Regarding the same improvement, another participant said, *“You were more, I guess, explicitly giving the constraints for what you wanted to optimise towards.”*

When comparing version A and B for Task 1, participants found that version A seemed like taking the planner role, and it was monotonous because of having to use multiple consecutive steps, while B only required one step. Participants said, *“It felt like, yeah, having to kind of create the plan yourself”* and *“Tedious to go through step by step.”* This indicates version B is supporting users to better shape the objectives of the mission.

*Information content of explanations* The participants appeared to find Task 2 relatively simple. It required keeping the asset away from a single point, and it was possible to complete the task using only one query in version A. However, they did not conclude anything about task performance since the explanation only showed time optimisation, saying, *“I feel like the situation is that the planner just simply doesn’t know [my objective], so it could say anything: say it got better; say it got worse. Either way, you have to go around the maintenance point.”*

Regarding version B, participants understood that they were changing the plan by changing the optimisation criteria, and that the explanation reflected this. Participants stated: *“I honestly, I would prefer the [version B explanation] in terms of giving me more information”*, *“So it’s much more useful”*, *“Yeah, that’s definitely better. Yeah, because this is information that you care about”*, *“So having the two different contrastive explanations are very useful.”*, and *“Yeah, it’s telling me that it will take longer, but it will be more efficient at carrying out the criteria that I’ve requested.”* This indicates version B allows users to get more useful information out of the explanations and better understand the trade-offs.

*Communication at an appropriate level* Participants found Task 3 difficult to solve using only queries in version A, because the original plan contained multiple points of communication loss. Once participants were presented with version B, in which they could use collections of objects as elements of the function and optimisation criteria, they quickly found the correct queries and solved the task in just two steps. In addition, they clearly showed more engagement with the system and the queries due to its new capabilities. In comparison between versions A and B, participants stated: *“So, [the version B queries] are closer to what I want to happen to the goal”*, *“[version B queries], they were conceptually simpler to kind of align with the overall goals”*, *“But just by using [the version B queries], it kind of gets me to where I want to be more or less very fast.”*, and *“I think the compound objects make it easier to get an overall plan. Within the constraints of your objectives.”* They also commented about version B, *“I think these kinds of queries are a lot easier, I would say: Fewer parameters or arguments”* and *“I could see them being quite simple to use.”* This indicates that version B is allowing users to convey their intent with more appropriate levels of abstraction.

*Alternative Strategies* As the participants got use to the system they suggested several interesting alternative strategies for tackling the tasks. A participant asked whether several questions together was possible: “Can you ask it to do multiple at once? I.e., why not have bluerov1 do [survey1] and [survey2] instead?” Although a similar effect can be achieved with an *‘Why A before B?* type query, the use of compound queries more generally is interesting. A participant asked: “Why is BlueROV1 not doing both survey zero and one?”, which can be considered as a question about resource allocation. The participants were not provided with the resource allocation queries in the study, which could have been used to support this sort of query. It was noted that sometimes the desired concept is not available, e.g., “what you want is for it to do the closer Objective, but you can’t explicitly just say give BlueROV2 to the closer objective.” In this specific case, after discussion it was decided that the problem was actually better conceptualised as a trade-off between objectives. However, this provides another example of how a (potential) MAST concept could be included and used by the user to communicate their intentions.

*User Feedback on the Interface* Several interesting ideas came up during the interviews, including query and interface level comments. It was noted that the previous plans, in terms of their metric valuations, should be immediately visible in the interface: “I think it will be just useful to have the criteria on the side to be able to compare each plan.” Several participants questioned whether the monitor step was necessary: “I do wonder why I couldn’t have jumped straight to [minimise] instead of having to [monitor] and then [minimise].” Finally, a participant questioned always using why queries: “You have to query in a very structured way; Could be quite annoying I guess.” We discussed an alternative to use ‘force *A* into plan’ directly.

### Discussion and future work

The collection of MAST concepts improved communication and understanding between participants and the planning model. Participants reported that it was easier to capture their strategy, suggesting the concepts more accurately reflected the users’ intentions. It also better supported their exploration of the planning space, and created explanations that were more meaningful for their task. These improvements contribute to bridging the gap in the human and agent models.

*Exploiting Common Structure in Planning Domains* The basic toolkit can be used with any planning domain, and the MAST concept wrappers can be used with MAST domains. The empirical evaluation demonstrated that the approach can be applied to other MAST domains, that concepts can be added, and used in queries, and that the system generates appropriate information (e.g., the trade-offs between metrics). This provides the first steps towards building a library of wrappers, which provide useful additional concepts and the machinery to automatically extend planning models to support those concepts. It demonstrates that through identifying an appropriate structure that the effort required to develop these wrappers can be shared across domains that share the structure.

Related model structures have been identified in planning models automatically (see Subsect. 2.2) [33]. This relies on domain model analysis, so that the analysis guarantees the appropriateness of the approach. It would be interesting to explore extending these approaches to the structures that we consider in this work, and specifically for use with temporal domains. This could support an automatic process for selecting the appropriate concept libraries for a planning domain.

*Using the Framework with Other XAIP Approaches* In this work, we have presented a toolkit for interpreting plans and extending planning models using MAST concepts. We instantiated these ideas within a specific XAIP approach (XAIP-as-a-service) and some aspects of the framework are tailored to this setting (e.g., plan comparison visualisation, numeric function queries).

However, we anticipate that elements of the framework are applicable beyond this particular approach, and moreover, that it provides a reusable template that could support a range of XAIP methodologies. For instance, in approaches that handle contrastive queries (e.g.,[82]), the middle-layer concepts can facilitate more effective communication, allowing users to better articulate the objectives they care about. Similarly, in approaches that learn a user’s important measures [83], the middle layer could enrich learnt representations with additional concepts, thereby supporting a more accurate characterisation of user intent.

The framework also has potential applicability in other XAIP paradigms. For example, [4] demonstrates how a similar structure can generate explanations for specific structures. In their approach, a navigation structure enables explanations for why an object cannot move between two disconnected points (e.g., its start and goal). In model reconciliation, the additional concepts could provide new ways of characterising and communicating model differences. For instance, if a user believed that two robots must always remain at least 10 metres apart, then equipping the system with the ability to represent and reason with such a constraint would allow it to generate more accurate explanations and more explicable plans.

*Adding New Concepts* Of course, there remain challenges in closing the gap between the human and agent representations. We considered a relatively small number of concepts. There are of course more concepts that could be supported within the MAST structure, including more multi-agent concepts, e.g., around coordination. During the study, participants suggested using alternative strategies and used language that suggested both additional terms that could be supported by the MAST structure (e.g., *‘closer’, ‘closest’, and ‘furthest’*). There are also other structures that could be considered, and used in order to build sets of concepts. We have considered resource allocation in this work, but there are other structures such as scheduling, or trajectory constraints, which might support additional important concepts. Further work is required to investigate the appropriate number of concepts, and whether there is a trade-off in terms of increasing the number of concepts, or increasing the complexity of the natural language processing mapping task. Our approach to increasing concepts has the advantage that the model is extended with the structure, which allows appropriate contrastive plans to be generated using the user’s queries.

*Relationship with LLMs* Although it was not appropriate to use LLMs in the context of the UAV scenario in this work (see Section 2), they provide interesting opportunities for the XAIP toolkit in the future. Although promising, LLMs are not currently able to generate fully operational planning models [84], and making error-free planning model extensions is also unlikely. However, there are interesting questions around supporting more general user interaction in the space of planning decisions, plans, state spaces, and plan spaces. For example, while doing the tasks in the study, several of the participants used context specific references to describe a situation (e.g., in the context of a specific state, referring to the task that was closest to the asset), and supporting this sort of analysis is an interesting challenge in this space. In this work we have investigated additional query types, including introducing queries over numeric functions, user input to add new concepts to the model, and queries about the resource allocation. This has allowed the user to impact on additional parts of the planning model, including adding new structure, and changing the optimisation criteria.

## Conclusion

In this work, we considered the problem of plan explanation and plan space exploration, in the context of underwater autonomous vehicle (UAV) missions. We proposed the multi-agent spatial-temporal (MAST) structure, which is a common structure in planning problems (and underpins our UAV scenarios). We demonstrated how this structure provides an interpretation of additional concepts related to the planning model, such as distance travelled, and proximity. We demonstrated how these concepts can be made available to the user through a collection of functions that are associated with automatic language extensions, which add new structure to the planning model. We then considered the problem of plan exploration, with an extension to the set of queries supported by XAIP-as-a-service. We provided an empirical evaluation that examines how these new query types behave in the context of the new structures. Finally, we summarised a qualitative user study, which investigated the use of the new user query types in UAV mission scenarios. Our results confirm previous work supporting contrastive explanations, and indicate that the additional concepts allow the user to better shape mission objectives, better communicate their intent to the system, and extract more useful information from the explanations.

## Data Availability

The datasets and materials used during the current study are available from the corresponding author upon reasonable request. We are also working towards making the XAIP toolkit publicly available.

## Appendix A: MAST Specifications for Domains

This appendix presents example MAST specification files for the driverlog and rovers domains.

## Footnotes

1 www.icaps-conference.org/index.php/Main/Competitions.

2 RASA open source: https://rasa.com/docs/rasa/
